# Effects of maoto (ma-huang-tang) on host lipid mediator and transcriptome signature in influenza virus infection

**DOI:** 10.1038/s41598-021-82707-1

**Published:** 2021-02-19

**Authors:** Akinori Nishi, Noriko Kaifuchi, Chika Shimobori, Katsuya Ohbuchi, Seiichi Iizuka, Aiko Sugiyama, Keisuke Ogura, Masahiro Yamamoto, Haruo Kuroki, Shigeki Nabeshima, Ayako Yachie, Yukiko Matsuoka, Hiroaki Kitano

**Affiliations:** 1 Tsumura Kampo Research Laboratories, Tsumura & Co., Ibaraki, Japan; 2 Sotobo Children’s Clinic, Medical Corporation Shigyo-No-Kai, Isumi, Chiba Japan; 3 General Medicine, Fukuoka University Hospital, Fukuoka, Japan; 4 The Systems Biology Institute, Shinagawa, Tokyo Japan

**Keywords:** Immunology, Infectious diseases, Influenza virus, Pharmacology

## Abstract

Maoto, a traditional kampo medicine, has been clinically prescribed for influenza infection and is reported to relieve symptoms and tissue damage. In this study, we evaluated the effects of maoto as an herbal multi-compound medicine on host responses in a mouse model of influenza infection. On the fifth day of oral administration to mice intranasally infected with influenza virus [A/PR/8/34 (H1N1)], maoto significantly improved survival rate, decreased viral titer, and ameliorated the infection-induced phenotype as compared with control mice. Analysis of the lung and plasma transcriptome and lipid mediator metabolite profile showed that maoto altered the profile of lipid mediators derived from ω-6 and ω-3 fatty acids to restore a normal state, and significantly up-regulated the expression of macrophage- and T-cell-related genes. Collectively, these results suggest that maoto regulates the host’s inflammatory response by altering the lipid mediator profile and thereby ameliorating the symptoms of influenza.

## Introduction

Epidemic infections of influenza cause half of million deaths every year. Elderly and individuals who have underlying diseases with immune system dysfunction are especially vulnerable to influenza and often develop severe symptoms^1,2^. Although several anti-influenza drugs targeting influenza viral factors have been developed, problems such as the emergence of strains with acquired drug resistance remain unsolved^3^. Thus, there is a need for more effective medications to treat influenza virus infection. From this perspective, drug interventions targeted at the host immune response are an attractive approach to suppress the influenza virus and mitigate viral damage without drug resistance.

Cytokines and lipid mediators are heavily involved in the host response to infection, and appropriate control of these factors is considered to be critical for suppressing the severity of the infection^4,5^. In the case of infectious diseases, the acute proinflammatory response plays a vital role in host defense by attacking the virus and inhibiting its replication. However, such inflammatory responses also damage tissues, and an excessive response is adversely harmful to the host. Thus, modulating anti-inflammatory effects has long been an important area of research. Recent studies have shown that specific lipid mediators appropriately regulate the inflammatory response and its resolution. Furthermore, the role of lipid mediators that promote the resolution of inflammation—in particular, specialized pro-resolving mediators (SPMs) such as protectins, maresins, and resolvins—have been the focus of many studies as endogenous modulators of the inflammatory response during infection^6–8^. The host response induced by influenza infection involves a complex interaction between the virus and several host factors^9^; thus, it might be appropriate to use a drug with a long tail to control this type of host response^10^. Furthermore, a traditional herbal medicine that contains multiple compounds might be an excellent option to simultaneously modulate the host factors.

Maoto (MT; or ma-huang-tang in Chinese), a traditional herbal medicine in Japan (Kampo), is prescribed widely to care for symptoms of upper respiratory infections and influenza^11,12^. Maoto is a mixture of four component herbs: Armeniacae semen (32.3%), Glycyrrhizae radix (9.6%), Cinnamomi cortex (25.8%), and Ephedrae herba (32.3%). The active ingredients for inflammation response in maoto such as amygdalin in Armeniacae semen, glycyrrhizin in Glycyrrhizae radix, which is metabolized to glycyrrhetinic acid and ephedrine in Ephedrae herba has been reported^13–15^. Furthermore, we revealed the profile of ingredients in maoto after oral treatment, and we estimated that many ingredients and metabolites of maoto containing ingredients such as pseudoephedrine, methylephedrine, prunasin, liquiritigenin and isoliquiritigenin are absorbed and detected in plasma^16^. Maoto has antipyretic^17,18^ and anti-malaise effects in children^19^, and improves flu symptoms with efficacy comparable to that of neuraminidase inhibitors in adults infected with influenza A virus^11,20^. Experimentally, MT has been shown to decrease viral titer and exert an antipyretic effect^21,22^, as well as to ameliorate influenza virus-induced pneumonia^23^. The herbal ingredients in MT directly inhibit influenza viral replication in vitro, and affect inflammatory responses both in vitro and in an in vivo animal model^14,24–33^. While these findings suggest that MT directly inhibits influenza virus infection and regulates the host inflammatory response as a multi-ingredient drug, its detailed mechanism of action has not been clarified.

Studies using in vivo rodent models have shown that MT broadly ameliorates the acute inflammatory response and injury, including both acute lung injury induced by cold/warm cycles of stress^34^ and asthma in ovalbumin-sensitized mice^35^. Our previous study revealed that MT significantly ameliorates flu-like symptoms induced by polyinosinic-polycytidylic acid (PIC; a Toll-like receptor 3 agonist) and decreases the pro-inflammatory cytokine response^16^. We also found that MT influences broad lipid mediator responses. In the acute phase at 2 h after treatment, MT affects the broad ω-3 fatty acid (FA)-derived lipid mediator response associated with anti-inflammatory and pro-resolution responses, and also modulates the acute production of prostaglandins and leukotrienes by PIC. Those findings suggested that MT acts via a specific mechanism to ameliorate acute infectious disease via host lipid mediator and inflammatory systems, specifically affecting anti-inflammatory and pro-resolving factors in the resolution phase of infection.

In this study, we first evaluated the ameliorative effect of MT on a mouse model of influenza virus infection. We then analyzed the lipid mediator and transcriptome profiles on the fifth day after infection to identify endogenous factors affected by MT and to elucidate its pharmacological properties towards influenza virus infection.

## Results

### Maoto reduced viral titer and ameliorated phenotypes induced by influenza virus infection

**Figure 1:**
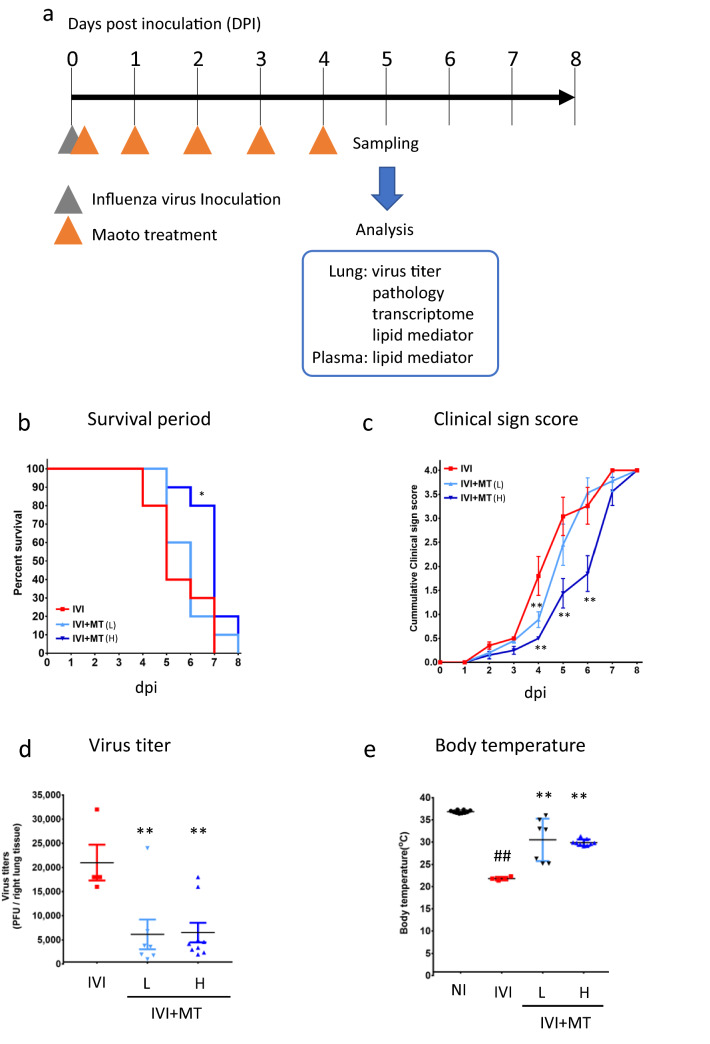

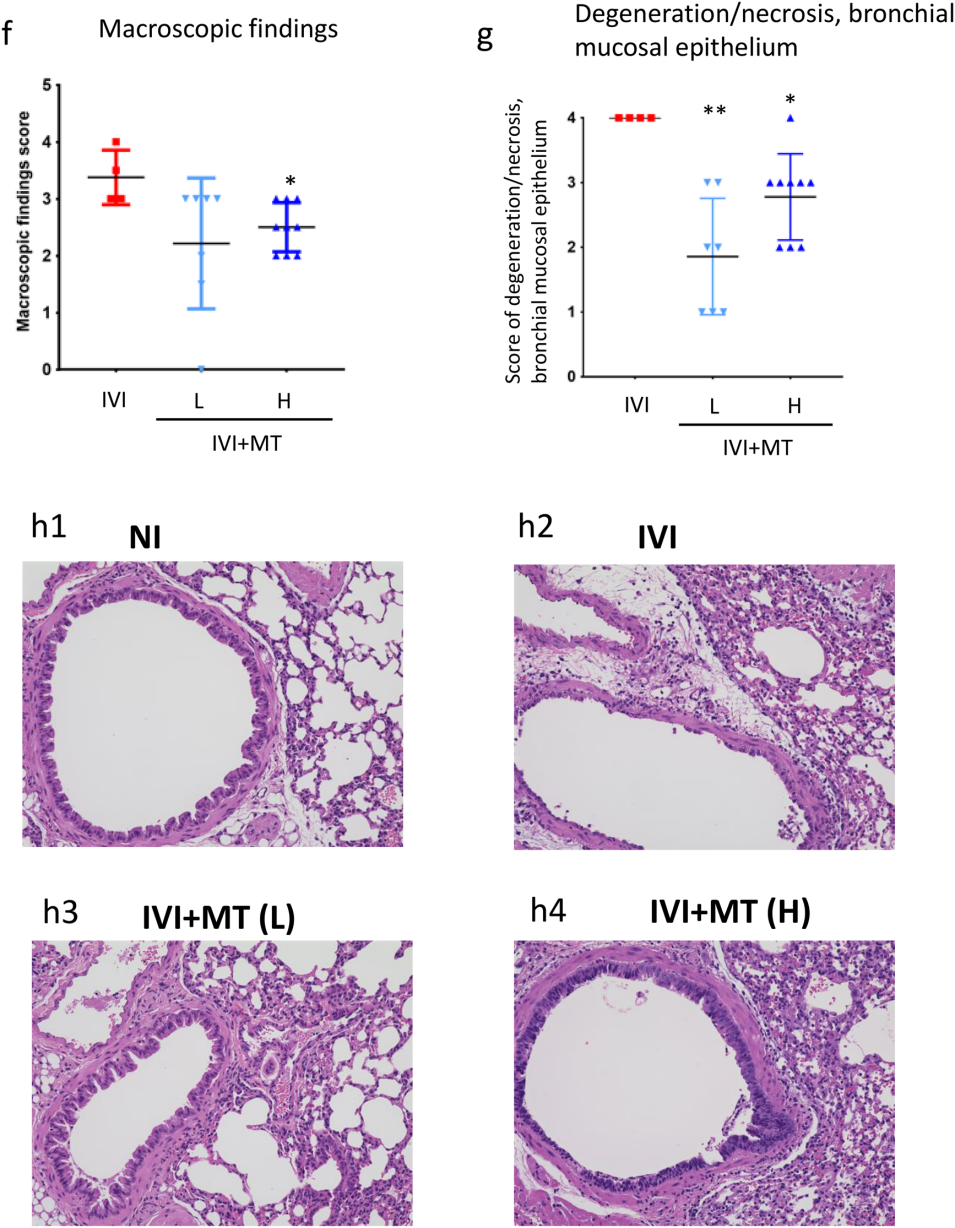
Effect of maoto on a mouse model of influenza virus infection. (**a**) Experimental protocol. Influenza virus was administered to mice by intranasal inoculation. Maoto (MT; 0.5 or 2 g/10 mL/kg) was orally administered 1 h after inoculation, and continued for 4 days (5 days treatment in total). Mice with no virus inoculation were used as a control against the infection, and infected mice treated distilled water were used as infected control mice. Data from 10 mice each in the influenza virus inoculation (IVI) control group, IVI with maoto 0.5 g/kg treatment [IVI + MT(L)] group, and IVI with maoto 2 g/kg treatment [IVI + MT(H)] group were used to determine the average survival period. Another 10 mice each in the no-inoculation (NI), IVI, IVI + MT(L), and IVI + MT(H) groups were sacrificed at 5 days post inoculation (dpi) to collect tissue and plasma samples for analysis of viral titer and histopathology. In addition, lipid mediator and transcriptome analysis were performed for the NI, IVI and IVI + MT(H) groups. At 5 dpi, the number of surviving mice were 10, 4, 7, and 9 in the NI, IVI, IVI + MT(L), and IVI + MT(H) groups, respectively. (**b**) Survival period (dpi). (**c**) Clinical sign score (dpi). (**d**) Viral titer. (**e**) Body temperature. (**f**) Macroscopic findings. (**g**) Histopathological score for degeneration and necrosis of bronchial mucosal epithelium. (**h**) Histopathological images of mouse lung tissue with hematoxylin–eosin staining (× 20). (**h1**) NI; (**h2**) IVI; (**h3**) IVI + MT(L); (**h4**) IVI + MT(H). * *P* < 0.01, ** *P* < 0.05 versus IVI by Log-rank test for survival period, by Mann–Whitney U test with Bonferroni’s multiple comparisons for clinical sign score, macroscopic findings, and histopathological score; and by Welch’s t-test with Bonferroni’s multiple comparisons for viral titer and body temperature.

The experimental protocol is shown in Fig. 1a. We infected mice with mouse-adapted influenza virus [A/PR/8/34(H1N1)] and then orally administered MT daily for 5 days starting 1 h after inoculation. The mortality and phenotype induced by the infection were observed for 8 days post inoculation (dpi).

The results showed that MT had anti-influenza virus activity. Whereas all mice in the influenza virus inoculation (IVI) group died by 7 dpi (median survival period, 5 days), those in the group with a higher dose of MT [IVI + MT(H)] lived for significantly longer (median survival period, 7 days) (Fig. 1b). The total clinical sign score [i.e., average summed score of four clinical signs (eye, fur, behavior, and other such as hypothermia, emaciation and respiratory failure), where individual signs were scored from 0 (normal) to 4 (death)], was also significantly reduced by MT treatment (Fig. 1c). The difference in survival rate between the IVI and IVI + MT(H) groups was most significant at 5 dpi. Therefore, we analyzed the effect of MT on viral titer and disease-induced phenotype (body temperature, body weight, lung injury, and pathological findings) at 5 dpi.

At 5 dpi, the number of surviving mice in each group was 4/10 (IVI), 7/10 [lower dose of MT, IVI + MT(L)], and 9/10 [IVI + MT(H)]. The survival rate was significantly higher for the IVI + MT(H) group than for the IVI group. The viral titer at 5 dpi was measured for all surviving mice. The IVI + MT(L) and IVI + MT(H) groups had significantly lower viral titers as compared with the IVI group (Fig. 1d). Regarding the disease-induced phenotype, MT administration significantly increased rectal temperature, which was significantly lowered by IVI (Fig. 1e), but there were no differences in body weight between the IVI and IVI + MT groups (Fig. S1). In addition, MT ameliorated infection-induced severe lung injury (Fig. 1f–h). The IVI + MT(H) group had significantly lower scores of macroscopic findings based on an evaluation of consolidation area from 0 (no consolidation) to 4 (consolidation area > 2/3) (Fig. 1f). Histopathological analysis showed that MT improved the degeneration and necrosis of bronchial mucosal epithelium induced by influenza infection (Fig. 1g,h; the recorded pathological scores are summarized in Table S1). Taking all observations of the phenotypic effects of MT into account, we used the high MT dose of 2 g/kg at 5 dpi as a measurement point for further analysis of host responses.

### Maoto modulated the host lipid mediator response

Based on the above effects of MT on the influenza viral titer and host phenotype, we analyzed lipid mediators in lung and plasma by LC–MS/MS in order to elucidate the host response in more detail. In total, the analysis covered 158 lipid mediators, including metabolites derived from ω-3 FA; docosahexaenoic acid (DHA) and eicosapentaenoic acid (EPA)-derived metabolites; and ω-6 FA-derived metabolites such as prostaglandins and leukotrienes (Table S2).

**Figure 2:**
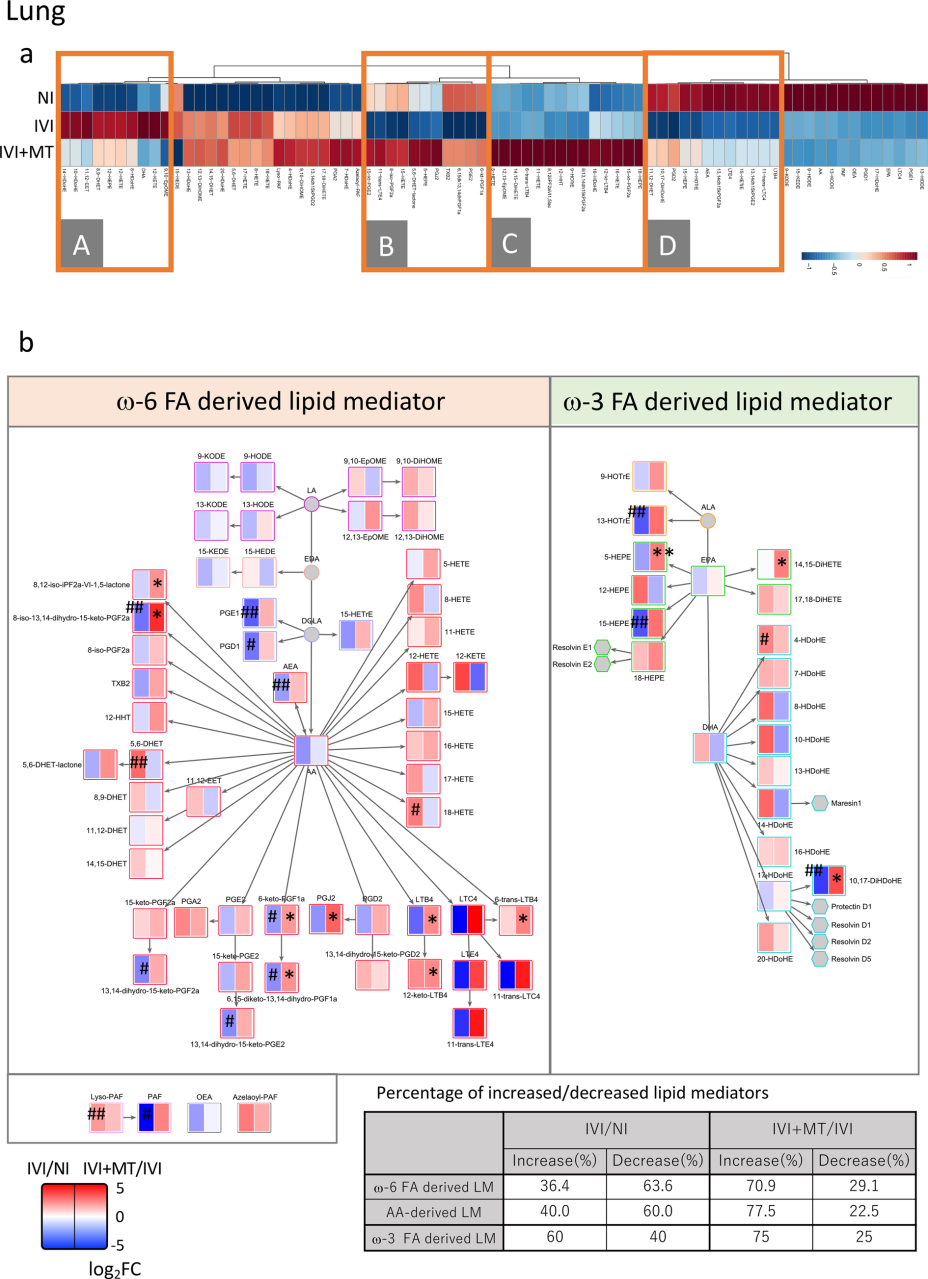

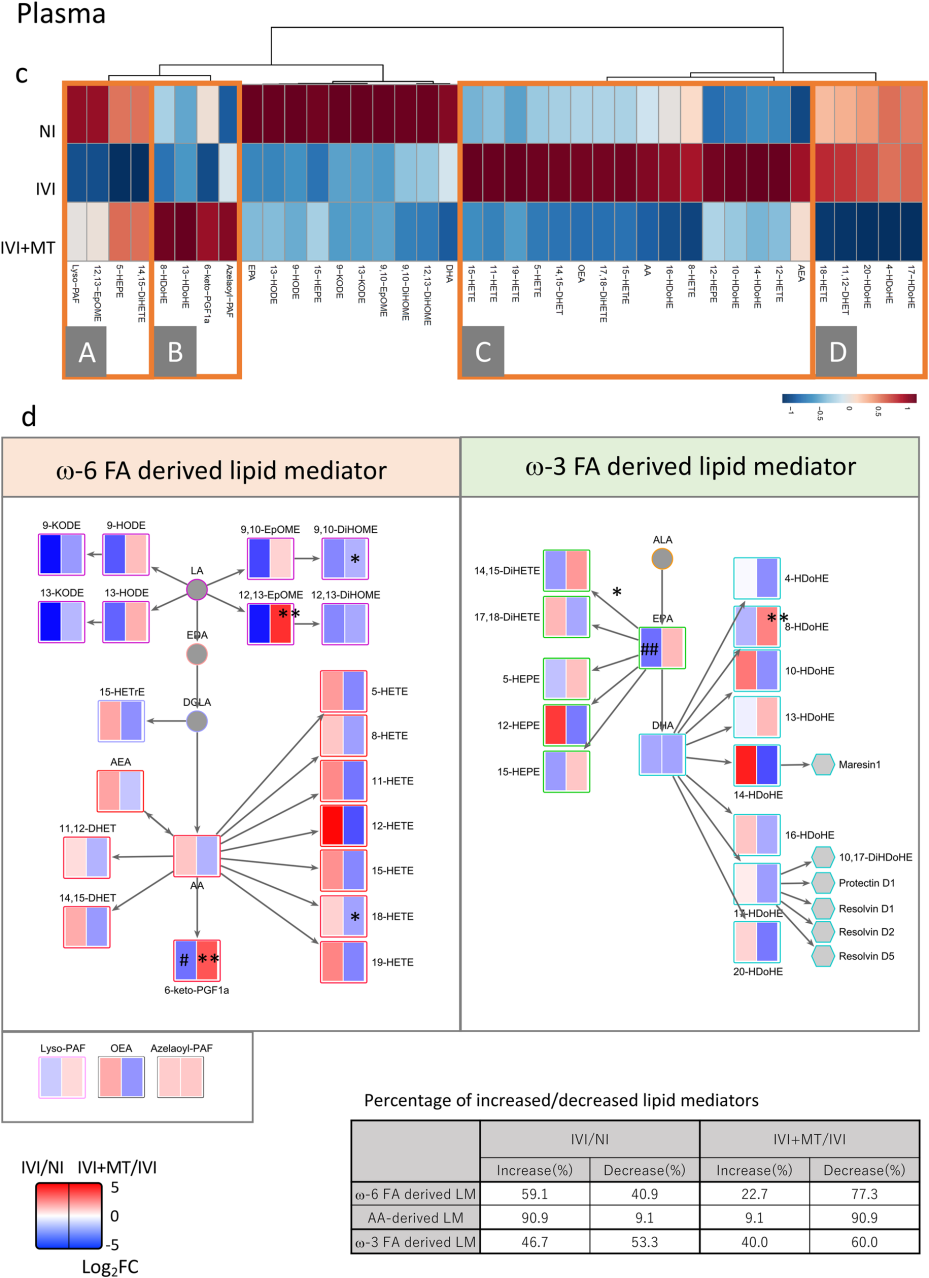
Clustering analysis and metabolic pathway mapping of lipid mediators in lung and plasma. (**a**) Clustering analysis in lung. A–D show specific clusters of lipid mediators that were altered by maoto (MT) relative to IVI. (**b**) Pathway mapping of lipid mediators in lung. Log_2_ fold changes (log_2_FC) in detected lipid mediators for IVI/NI (left column) and IVI + MT/IVI (right column) were mapped on the metabolic pathway. The percentage of increased or decreased lipid mediators for IVI/NI and IVI + MT/IVI is summarized in the table. (**c**) Clustering analysis in plasma. A–D show the specific cluster of lipid mediators that were altered by MT relative to IVI. (**d**) Pathway mapping of lipid mediators in plasma. The log_2_FC in detected lipid mediators for IVI/NI (left column) and IVI + MT/IVI (right column) were mapped on the metabolic pathway. To show the related pathway of specialized pro-resolving mediators (SPMs), undetected SPMs were included on the pathway as hexagons. The percentage of increased or decreased lipid mediators for IVI/NI and IVI + MT/IVI is summarized in the table. For clustering analysis, the data were normalized and standardized by autoscale for metabolites. Euclidean distance was used for the distance measure, and Ward’s method was used for the clustering algorithm. *P < 0.05, **P < 0.01 versus IVI. ^##^*P* < 0.01 versus NI. *P < 0.05, **P < 0.01 versus IVI. ^#^*P* < 0.05, ^##^P < 0.01 versus NI.

Overall, 77 lipid mediators were detected in lung. Clustering analysis showed that specific clusters were associated with MT (Fig. 2a and Fig. S2a). Specifically, lipid mediators in clusters A, B and D, which were increased (A) or decreased (B and D) by IVI, were normalized by MT administration. Furthermore, lipid mediators in cluster C were specifically increased by MT. The lipid mediators that were significantly affected by MT are shown in Fig. S2b.

Pathway enrichment analysis of the lipid mediators affected by IVI showed that IVI affected the balance of ω-6 and ω-3 FA-derived lipid mediators. In detail, 63.6% of ω-6 FA-derived lipid mediators and 60.0% of arachidonic acid (AA)-derived lipid mediators were reduced, and 60.0% of ω-3 FA-derived lipid mediators were increased by IVI (Fig. 2b and Fig. S2c). In comparison with the normal state, ω-3 FA-derived lipid mediators suggested activated. By contrast, MT tended to increase both ω-6 and ω-3 FA-derived lipid mediators: specifically, 70.9% of ω-6 FA-derived lipid mediators, 77.5% of AA-derived lipid mediators, and 75% of ω-3 FA-derived lipid mediators were increased by MT. Therefore, MT increased both ω-3 and ω-6 FA-derived lipid mediators, and enhanced the broad lipid mediator pathway in infected mice. This effect of MT seemed to be associated with both activation of the inflammatory response against the influenza virus and resolution of lung damage. Notably, in the lung, MT significantly increased metabolites in the pro-inflammatory pathway of leukotriene B4 (LTB4), including LTB4 and its metabolites 12-keto-LTB4, as well as prostaglandin J2 (PGJ2), which is involved in protecting the influenza virus infection-associated host immune response^5,36,37^. Furthermore, an ω-3 FA lipid mediator, 10(s),17(s)-dihydroxy-docosahexaenoic acid (10,17-DiHDoHE), which is associated with improvement of acute lung injury in IVI^38–40^, was significantly decreased by IVI and increased by MT. Therefore, MT enhanced host lipid mediators associated with decreasing influenza virus and with recovery of inflammation-induced tissue damage in lung.

Analysis of the lipid mediator profile in plasma detected 39 lipid mediators, which we subjected to cluster analysis to identify characteristics due to IVI and MT (Fig. 2c and Fig. S3a). Lipid mediators in clusters A and C were decreased and increased, respectively, by IVI, and were normalized by MT. The lipid mediators that were significantly normalized by MT are shown in Fig. S3b. Furthermore, lipid mediators in clusters B and D were specifically increased and decreased, respectively, by MT. In pathway enrichment analysis, 90.9% of AA-derived lipid mediators were increased by IVI. In infected mice, MT tended to reduce ω-6 FA-derived lipid mediators, especially AA-derived lipid mediators, and slightly reduced ω-3 FA-derived lipid mediators. In total, 77.3% of ω-6 FA-derived lipid mediators, 90.9% of AA-derived lipid mediators, and 60% of ω-3 FA-derived lipid mediators were reduced by MT (Fig. 2d, and Fig. S3c). Overall, systemic AA-derived lipid mediators tended to be reduced by MT, which suggests that the effect of MT on host lipid mediators in plasma may act to ameliorate the acute systemic inflammatory response.

### Maoto affected specific gene expression profiles associated with macrophage and T cell immune responses

Our analysis of the host phenotype and endophenotype for lipid mediators clearly showed that MT acts on the inflammatory immune response of the host. We therefore performed transcriptome analysis to assess the specific gene networks associated with IVI and MT. Weighted gene co-expression network analysis (WGCNA) was used to find the gene networks associated with NI, IVI, and IVI + MT (Fig. S4). In total, we identified 45 modules, among which 9, 17, and 12 modules were significantly associated with NI, IVI and IVI + MT, respectively (Fig. S4e).

**Figure 3:**
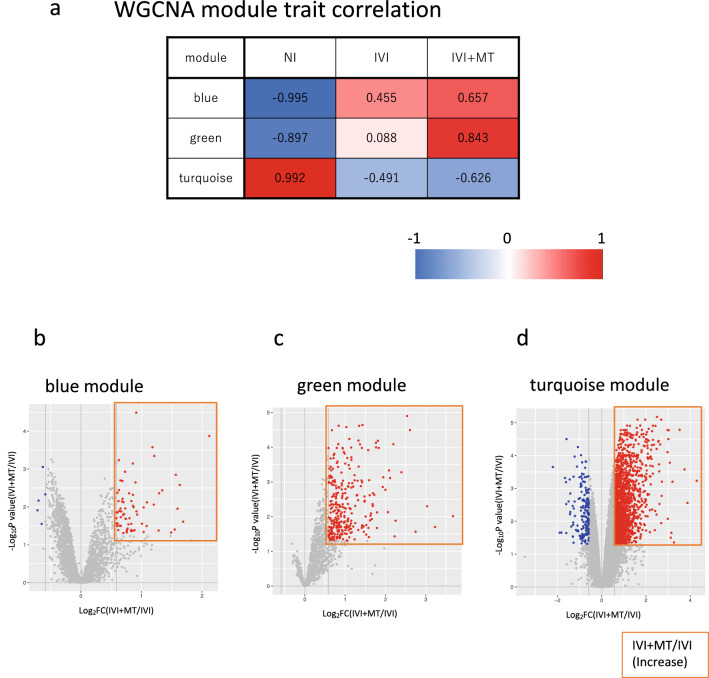

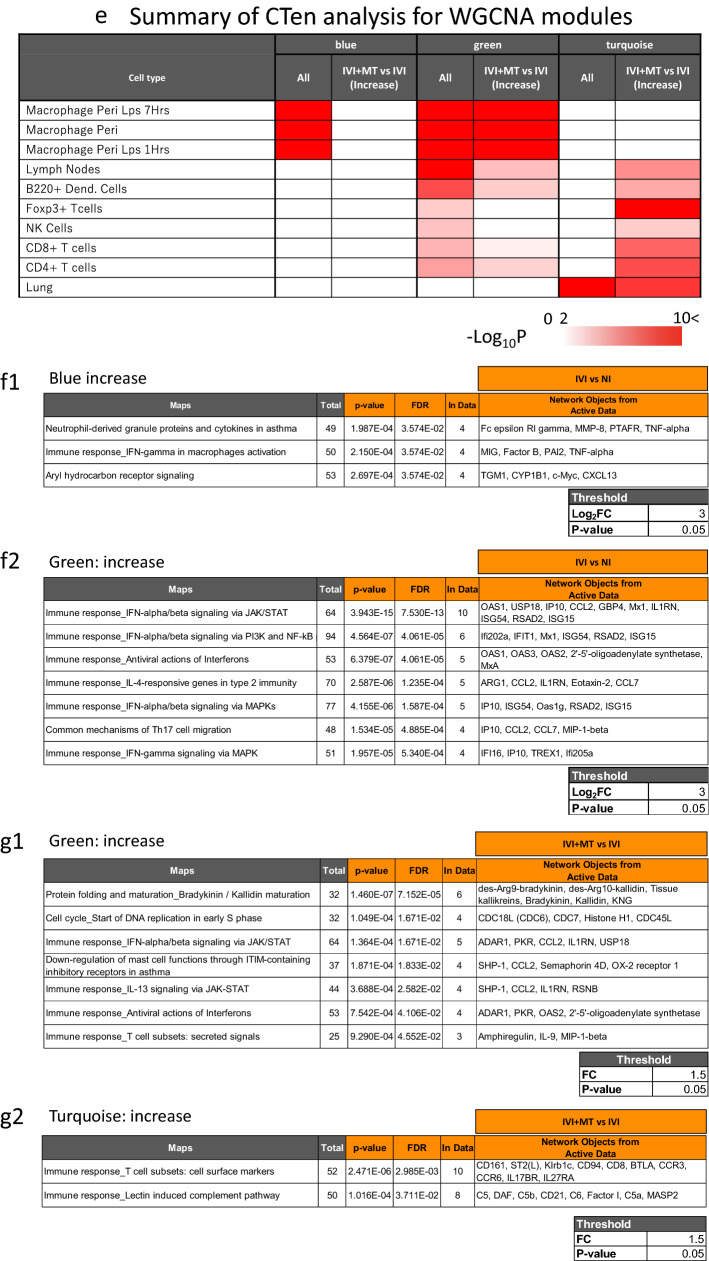
Weighted gene co-expression network analysis (WGCNA). (**a**) Correlation between module and trait. (**b**–**d**) Volcano plot for comparing gene expression between IVI + MT and IVI in the blue, green, and turquoise modules. Blue indicates a significant decrease in gene expression in IVI + MT relative to IVI at *P* < 0.05 and fold change (FC) < 0.67; red indicates a significant increase in gene expression in IVI + MT relative to IVI at *P* < 0.05 and FC > 1.5. (**e**) Summary of cell type enrichment analysis (CTen). –log_10_ Benjamini–Hochberg adjusted *P* > 2 was considered to be significant. For each module, the left column shows the results using all genes in the module; the right column shows the results using genes significantly increased by MT relative to IVI. (**f**) Pathway enrichment analysis of differentially expressed genes between IVI and NI in the blue (FDR < 0.05) (**f1**) and green (major top 7 pathways with FDR < 0.05) (**f2**) modules. The gene sets significantly increased by IVI relative to NI at criteria of *P* < 0.05 and log_2_FC > 3 were used for analysis. (**g**) Pathway enrichment analysis of differentially expressed genes between IVI + MT and IVI in the green (FDR < 0.05) (**g1**) and turquoise (FDR < 0.05) (**g2**) modules. The gene sets significantly increased by MT relative to IVI at criteria of *P* < 0.05 and FC > 1.5 were used for analysis.

Next, we conducted cell-type enrichment (CTen) analysis on the WGCNA-identified modules, which revealed that eight modules were significantly associated with specific cell types (Supplementary Fig. S4f). We also found that three modules (colored-coded as blue, turquoise and green) including relatively large gene sets (blue, 2917; turquoise, 9504; green, 1220), which were significantly enriched in the immune cell marker genes defined in CTen, were correlated with MT, and showed a specific pattern of correlation with each trait (Fig. 3a and Figs. S4g-i). The blue module negatively correlated with NI and positively correlated with IVI and IVI + MT; the turquoise module positively correlated with NI and negatively correlated with IVI and IVI + MT; and the green module negatively correlated with NI, positively correlated with IVI + MT, but did not correlate with IVI. Furthermore, we observed gene sets that were significantly increased/decreased by MT in each module (Fig. 3b–d).

To identify specific gene functions within these three modules, we analyzed the cell type specificity induced by infection and MT by using CTen analysis. Based on all genes in each module (Fig. 3e and Fig. S4j), CTen analysis showed that the blue module was mainly enriched in macrophage cell types, the green module was broadly enriched in many kinds of cell type, while the turquoise module was enriched in lung cell types. These results showed that IVI induces a broad host immune response, such as migration/activation of macrophages, and disrupts the normal profile of lung cell gene expression at 5 dpi. By contrast, the genes increased by MT in the blue and green modules were especially enriched in macrophage cell types, while those increased by MT in the turquoise module were mainly enriched in T-cell types. These results suggest that MT notably enhances macrophage and T-cell immune responses in influenza infection (Fig. 3e and Fig. S4j).

Using MetaCore, we conducted pathway enrichment analysis to reveal the module functions associated with influenza infection and MT treatment. This analysis showed that the genes significantly increased by IVI relative to NI (blue and green modules) were mainly enriched in the interferon-γ-associated macrophage activation (blue), neutrophil-derived granule protein and cytokine (blue), aryl hydrocarbon receptor signaling (blue), and interferon signaling (green) pathways (Fig. 3f). On the contrary, no enriched function was observed among the genes with increased expression in the turquoise module (Fig. 3f). Collectively, the results of cell type and pathway enrichment analysis indicated that host responses involving interferons and cytokines in macrophages and neutrophils, which are closely associated with the acute host response in influenza virus infection^41,42^, continued in inoculated mice until 5 dpi.

We then evaluated the effect of MT on these modules. The genes that were significantly increased by MT relative to IVI (green module) were mainly enriched in bradykinin and kallistatin maturation, and in inflammatory responses such as interferon signaling (Fig. 3g1). In addition, the genes that were significantly increased by MT in the turquoise module were primarily enriched in the immune response of the T-cell subset and lectin-induced complement pathway (Fig. 3g2). Furthermore, the gene expression of amphiregulin, which is associated with tissue repair by macrophage and innate lymphoid cells^43^, was significantly increased by MT [log_2_FC(IVI + MT/IVI) = 0.858, *P* < 0.01]. These WGCNA-based data were consistent with the findings from the analysis of differentially expressed genes (DEG) between IVI and NI, and between IVI + MT and IVI (Fig. S5). Notably, key factors such as interferons, lipid mediators, and amphiregulin were increased by MT, which indicates that MT might enhance host responses against influenza virus infection and aid recovery from severe lung tissue damage.

**Figure 4:**
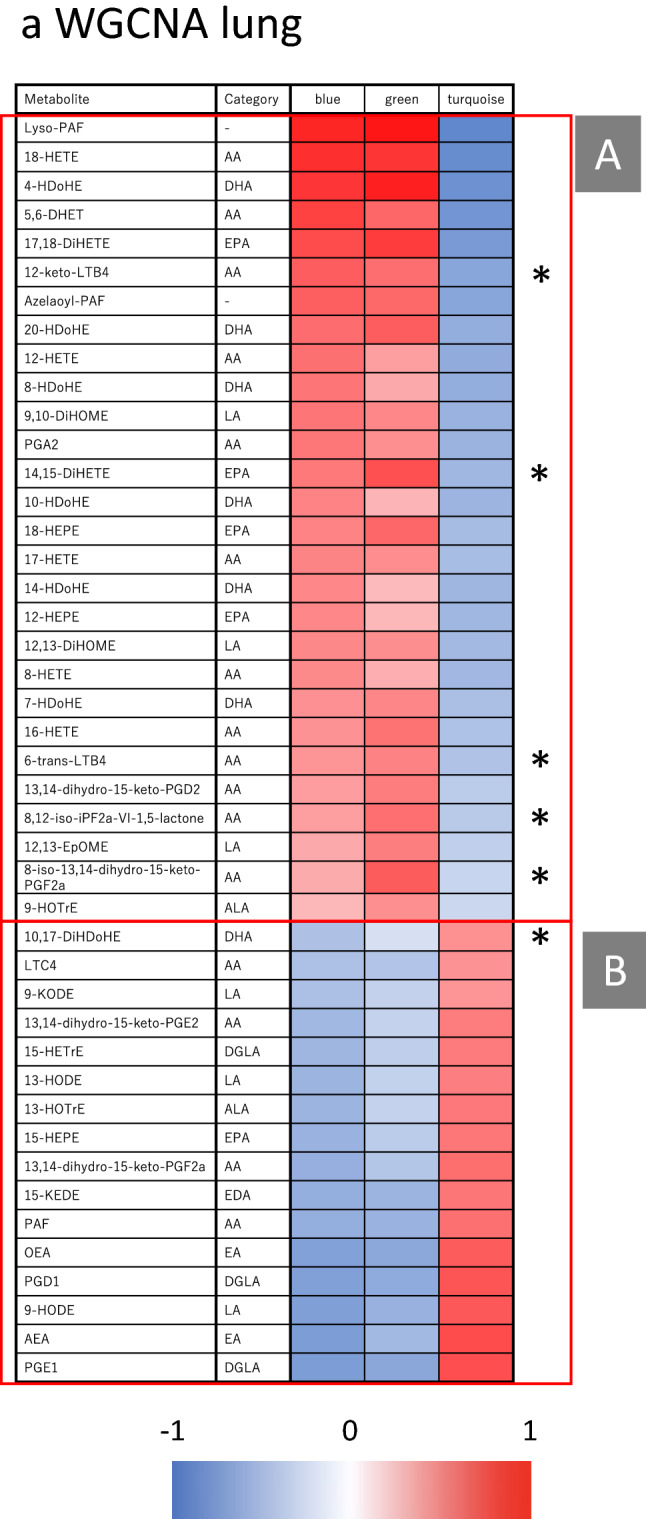

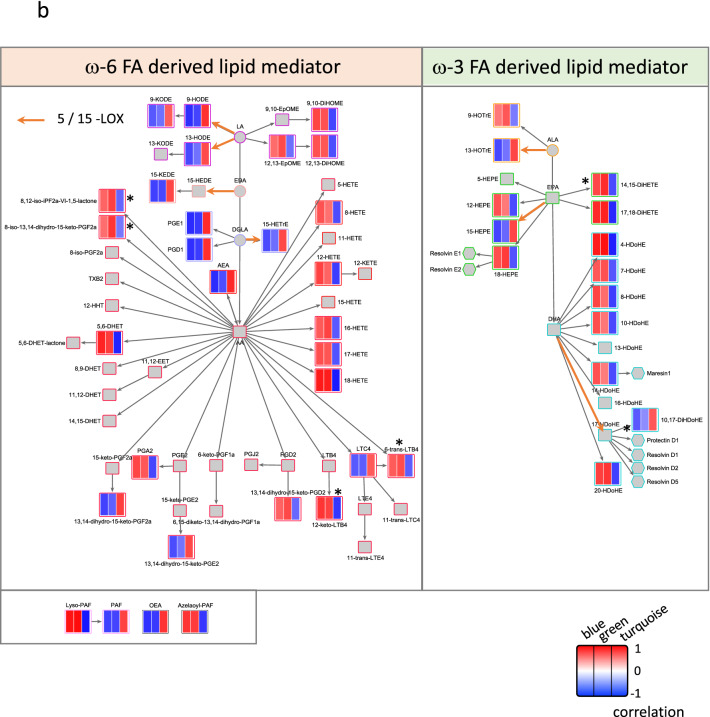
Correlation between gene expression networks and the lipid mediator profile. (**a**) Correlation between modules detected by WGCNA (blue, green, and turquoise) and lipid mediator profile. Correlations between the gene expression profile and the lipid mediator profile, which was used as trait, were analyzed by WGCNA. Asterisks indicate lipid mediators that were significantly altered by MT relative to IVI. (**b**) Pathway mapping of lipid mediators in lung. The WGCNA correlation score of the blue (left column), green (middle column), and turquoise (right column) modules of detected lipid mediators were mapped on the metabolic pathway. Lipid mediators that were detected but not included in the WGCNA modules are indicated by small grey-filled squares. *P < 0.05 for IVI + MT versus IVI by Welch’s t-test with Bonferroni’s multiple comparisons.

Lastly, we assessed the correlations between gene expression and lipid mediator profiles by WGCNA. For this analysis, the intensity of lipid mediators was used as the trait. As a result, we found lipid mediators that were significantly correlated with the blue, green and turquoise modules (Fig. 4a). The blue and green modules showed a similar correlation pattern, whereas the turquoise module showed an opposite pattern. We then assessed the correlation patterns of the lipid mediators within their metabolic pathways (Fig. 4b). On the one hand, lipid mediators derived from AA and DHA tended to be positively correlated with the blue and green modules. Because these two modules were significantly associated with macrophage gene sets, the lipid mediators positively correlated with these modules may be associated with macrophage function. On the other hand, several lipid mediators metabolized by 5-lipoxygenase (5-LOX) or 15-LOX, 13-hydroxy-octadecatrienoic acid (13-HOTrE), 10,17-DiHDoHE, 15-hydroxy-eicosapentaenoic acid (15-HEPE), 9-hydroxy-octadecadienoic acid (9-HODE), 9-oxo-octadecadienoic acid (9-KODE), 13-HODE, and 15-hydroxy-eicosatrienoic acid (15-HETrE) were positively correlated with the turquoise module. Because the turquoise module is enriched in lung functions, metabolites in the 5- and 15-LOX pathway may be potentially associated with lung tissue function. Furthermore, 12-keto-LTB4, 6-trans-LTB4, 8,12-iso-isoprostane F2 α-VI-1,5-lactone (8,12-iso-iPF2a-VI-1,5-lactone), 8-iso-13,14-dihydro-15-keto-PGF2a, and 14,15-dihydroxy-eicosatetraenoic acid (14,15-DiHETE) were significantly increased by MT relative to IVI and positively correlated with the blue and green modules. Thus, these lipid mediators might be associated with macrophage cells induced by MT.

## Discussion

Herein, we have confirmed that MT has anti-influenza virus activity and reduces viral titer, consistent with previous studies^21–23^. We have extended previous research, conducting a more comprehensive analysis of the effect of MT on the host response induced by IVI by examining lipid mediator and gene expression profiles at 5 dpi in a mouse model of IVI with MT treatment as summarized in Table 1.

**Table 1:**
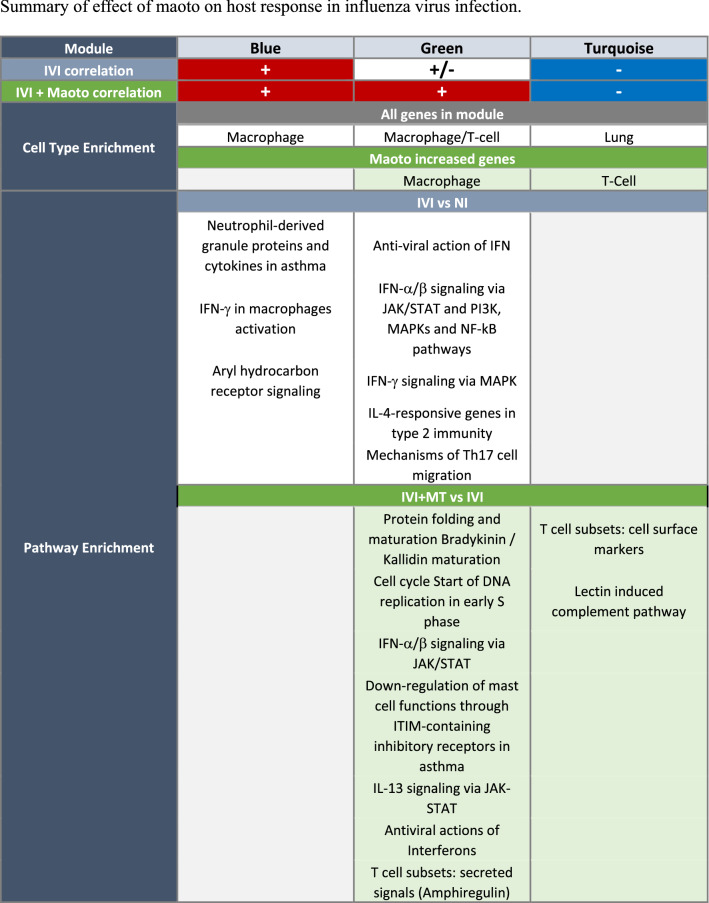
Summary of effect of maoto on host response in influenza virus infection.

Lipid mediators are associated with and ameliorate pathogenesis in IVI^4,5^. For example, Tam et al.^4^ previously examined the changes in lipid mediators in bronchoalveolar lavage during IVI, showing that a lethal infection resulted in an increase in ω-3 FA-derived lipid mediators at 5 dpi, which is consistent with the trends observed in our data. We further showed that MT tended to increase these mediators. Because ω-3 FA-derived lipid mediators generally play a role in modulating inflammation, the enhanced response of the ω-3 FA-derived lipid mediators in the infected state may be part of the host defense response to infection. Therefore, these observations may suggest that MT improves the lipid mediator profile, and this endophenotype seems to correlate with a prolonged survival period.

Our analysis of lipid mediator profiles in lung further identified two types of responses of lipid mediators associated with MT treatment. The first type of response, which involved lipid mediators normalized by MT during the infection, was associated with the IVI-induced immune response (cluster A, B and D in Fig. 2a). The second type of response was associated with an MT-induced specific immune response (cluster C in Fig. 2a). Several lipid mediators increased by MT at 5 dpi were located on pathways involved in the immune response to protect against and reduce influenza viral load, such as LTB4^36,44^, which regulates type 1 interferon signaling and interstitial macrophages^36^, and PGJ2 as a source of 15-deoxy-delta-13,14-PGJ2^37^. Furthermore, 10,17-DiHDoHE (also known as PDX or protectin D1) improves severe influenza virus infection, bleomycin-induced lung dysfunction and lipopolysaccharide-induced acute lung injury^5,38,39^. Thus, MT seems to enhance pathways associated with both specific proinflammatory factors and SPMs, which contribute to protection against IVI and resolution of inflammation. Lipid mediators are known to switch class as acute inflammation progresses to resolution. In the early stages of acute inflammation, lipid mediators activate neutrophils to promote phagocytosis, but then switch to recruitment of macrophages. PGE2 and PGD2, which tended to be increased by MT, ultimately activate resolution-type lipid mediators such as lipoxins, resolvins, and protectins, leading to the elimination of inflammation^8^. Although not all resolution-type lipid mediators were identified, our finding that MT increased 10,17-DiHDoHE at 5 dpi suggests that MT enhances the transition from acute inflammation to resolution. Maintaining the augmentation of lipid mediators may accelerate recovery. Our previous study found that MT is a crude drug mixture that alters many lipid mediators upon intake. Such augmentation of the dynamics of the lipid mediator profile may contribute to the relief of symptoms.

Regarding the lipid mediator profile in plasma, we observed that IVI caused an increase in AA-derived lipid mediators, which was then reduced by MT treatment at 5 dpi. Because these lipid mediators are generally known as inflammatory factors, MT might suppress systemic inflammation by suppressing such lipid mediators. Several lipid mediators were significantly increased by MT, including 12,13-epoxy-octadecenoic acid (12,13-EpOME), 6-keto-PGF1α, 14,15-dihydroxy-eicosatetraenoic acid (14,15-DiHETE), and 8-hydroxy-docosahexaenoic acid (8-HDoHE), while those decreased by MT were 9,10-dihydroxy-octadecenoic acid (9,10-DiHOME) and 18-hydroxyeicosatetraenoic acids (18-HETE). Although the causes of these alterations by MT are unclear, the lipid mediators identified in plasma may be useful as biomarkers of the MT effect.

In our previous study using a rat model of PIC**-**induced acute inflammation, the prostaglandins and leukotrienes induced in plasma were reduced by MT at 2 h after treatment, suggesting that MT alters the host inflammatory response at an early phase of infection. Because the PIC injection model is considered to partially mimic the host response to virus, it is possible that MT might regulate the host response profile in IVI in a similar manner. That is, in the early phase of viral infection, MT may affect the systemic lipid mediator response for prostaglandins and leukotrienes. Subsequently, in the sub-acute or late phase of the infection, it may modulate the broader ω-6 and ω-3 FA-derived lipid mediator response, providing a comprehensive host immune response and intervening in viral replication. In this study, we only examined the effects at 5 dpi; therefore, we can only hypothesize about the dynamics of the switch in lipid mediator class reflecting the intervention of MT in the host response.

In the transcriptome analysis, gene expression associated with type I interferon, neutrophils and macrophages was induced by influenza infection, consistent with a previous study on transcriptome analysis in IVI^45^. In the early phase of infection, the phagocytotic activity of macrophages is essential to reduce the virus, and subsequently tissue-resident macrophages play a role in tissue repair. However, prolonged activation of macrophages derived from circulating monocytes promotes alveolar injury^46^. Our data suggest that the proinflammatory response induced by infection and the enhanced inflammatory response associated with acute lung injury were maintained until 5 dpi.

Cell type enrichment analysis showed that MT upregulated specific genes associated with macrophages and T cells, many of which seemed to be involved in migration and activation. This finding corresponds with that of a previous study of MT (ma huang tang) on H1N1 infection, which showed that MT significantly improved infection-induced lung injury, significantly decreased CD4^+^ and CD8^+^ T cells, and increased the ratio of CD4^+^/CD8^+^ T cells^23^. Although the immune cell population needs to be confirmed, these results suggest that MT modulates macrophage and T-cell dynamics, ameliorates the pathology induced by IVI, and repairs lung tissue.

In addition to the interferons and cytokines that MT modulates, we found that MT affect the gene networks for bradykinin and kallistatin maturation. In this pathway, tissue kallikrein is a vital molecule associated with regulation of alveolar macrophages and protection against influenza virus infection^47^. This pathway would have an essential role in the ameliorative effect of MT.

Furthermore, we found a correlation between the IVI-induced and MT-affected gene networks and lipid mediator response, whereby AA-derived lipid mediators and DHA-derived lipid mediators were positively correlated with the immune response induced by the viral infection, and lipid mediators in the 5-LOX or 15-LOX pathway associated with lung tissue-specific genes tended to be decreased by infection and improved by MT. This result suggests that the dynamics of the lipid mediator profile may reflect an improvement in the pathology. In particular, 12-keto-LTB4, which was positively correlated with a macrophage cell type by CTen, was significantly increased by MT (Fig. 4). Parnet et al.^36^ previously showed the LTB4 is closely associated with type 1 interferon and alveolar macrophage function, which is consistent with our lipid mediator and gene network analysis of MT. This may infer that MT enhances the LTB4 pathway and affects alveolar macrophage function and the associated gene network pathways such as type I interferon, before shifting the lipid mediator class towards pro-resolution, which leads to its ameliorative effects.

On the other hand, 10,17-DiHDoHE was correlated with the T-cell enriched module, which suggests that 10,17-DiHDoHE may be associated with the effects of MT via the T-cell response and tissue repair. The role of SPMs such as 10,17-DiHDoHE, which was increased by MT, has received much attention^8^. Furthermore, the metabolic pathway from DHA to 17-HDoHE is closely associated with the production of not only 10,17-DiHDoHE but also other SPMs, resolvins, although the latter lipids were not detected in the present study. These results suggest that MT may tilt the balance towards the pro-resolving phase and accelerate the recovery period in influenza infection by regulating the immune system.

In summary, we have shown that MT has the potential to ameliorate influenza virus infection by altering specific host responses. By modulating the host immune response, MT might be an effective treatment for influenza virus infection that would be free from the problems of drug resistance, unlike other antiviral drugs that directly target viral proteins. In our integrated analysis of lipid mediators and gene expression, along with cell type analysis, the group of genes associated with lipid mediators suggested that MT influences the activity of specific immune cells. Thus, the overall effect of MT on host lipid mediator response might be a synergistic action between anti-virus activity and amelioration of the systemic damage due to IVI. In particular, our results regarding the lipid mediators and immune cell groups induced upon infection confirm the observations of previous studies.

Zheng et al. previously reported that the combination of a neuraminidase inhibitor (zanamivir), cyclooxygenase-2 (COX-2) inhibitor (celecoxib), and anti-inflammatory drug (mesalazine) led to an improvement in lethality as compared with a single treatment of zanamivir in cases of IVI with delayed initiation of treatment^48^. While it will be important to clarify the dynamics of the effect of MT on the host response against IVI in future research, MT affects multiple targets and ameliorates the symptoms of influenza as multi-compound herbal medicine. In addition, the study identified lipid mediators in plasma, which may be potential candidates as a biomarker to verify the effect of crude drug administration, once the connection between lipid mediator profiles in lung and plasma is confirmed. Lastly, although our study confirmed the effect of MT administration on the 5th-day post-infection, there are some limitations of this study. While we elucidated dynamic alteration of gene expression associated with immune response, we further need to reveal the details of immune cells affected by maoto. The mechanism of action of maoto that triggers the host response during the initial administration remains to be evaluated by examining a time-dependent analysis of host responses against IVI coupled with MT treatment. Given that maoto is a multi-compound herbal medicine, it is vital to elucidate the synergetic effect of active ingredients in maoto to reveal the specific mechanism of action of maoto as a long-tail drug. Finally, we should integrate the response of the complex host, virus and multi-compound response using systems biology and network pharmacology^10,49–51^. These approaches have been applied to reveal the complex interaction between endogenous factors and multi-compound of traditional medicines for kinds of disease such as influenza, cancer, type 2 diabetes and rheumatoid arthritis^52–56^. Applying the approach is valuable to elucidate the overall mode of action of maoto and for repositioning of maoto to related disease. Furthermore, understanding how maoto modulates host inflammation response and immune response gives essential information not only for the anti-influenza virus activity but also therapeutic potential against other acute infectious diseases, and excessive inflammatory response such as cytokine storm.

## Methods

The detailed methods were shown in supplementary methods.

### Materials

Maoto is an extracted mixture of Ephedrae Herba (32.3%), Armeniacae Semen (32.3%), Cinnamomi Cortex (25.8%), and Glycyrrhizae Radix (9.6%). Dry powdered extracts of MT were supplied by Tsumura & Co. (Tokyo, Japan). Maoto was dissolved in distilled water before administration to mice.

### Virus culture

Mouse-adapted influenza virus [A/PR/8/34(H1N1)] (PR8) was used to infect mice. The influenza virus was cultured in Madin–Darby canine kidney (MDCK) cells, and PR8 collected from the culture medium was used for intranasal inoculation of mice.

### Animals

Female BALB/c Cr Slc mice purchased from Japan SLC, Inc. (Shizuoka, Japan) were used from 6 weeks of age after habituation. The mice were housed in groups under the following conditions: room temperature, 19–24 °C, relative humidity, 40–70%, and 12-h light–dark cycle (6:00–18:00). All experiments were approved by the Laboratory Animal Committee of Tsumura & Co. and Nihon Bioresearch Inc. (Hashima, Gifu, Japan), and performed in accordance with guidelines for the conduct of animal experiments in ministry of health, labour and welfare, Japan.

### In vivo influenza virus inoculation

Influenza virus (1 × 10^5^ PFU/0.05 mL/mouse) was administered by intranasal inoculation. Maoto (0.5 or 2 g/10 mL/kg) was orally administered for 5 days starting 1 h after inoculation. Mice with no inoculation were used as control mice against the infection, and infected mice that were treated distilled water were used as infected control mice. In total, there were four groups of mice: (1) no-inoculation (NI) group; (2) influenza virus inoculation (IVI) control group; (3) IVI with MT 0.5 g/10 mL/kg treatment [IVI + MT(L)] group; and (4) IVI with MT 2 g/10 mL/kg treatment [IVI + MT(H)] group. Daily body weight and clinical signs of each mouse were recorded. Clinical signs were divided into four categories, eye, fur, behavior and other (hypothermia, emaciation and respiratory failure), which were individually scored from 0 (normal) to 4 (death). The scores of the four categories were averaged per treatment group and the total clinical sign score was obtained. Rectal temperature was measured by using a thermometer (Physitemp, Model BAT-12, Physitemp Instruments Inc.).

Ten mice from each of the four mouse groups were used to record the survival period. Another 10 mice in each group were sacrificed at 5 dpi to collect tissue and plasma samples for analysis of viral titer, histopathological findings, transcriptome analysis, and lipid mediator analysis.

### Tissue sampling

Lung tissues and plasma were collected at 5 dpi. Blood was collected and the plasma sample was stored at –80 °C. The lung tissue was scored for macroscopic findings by area of consolidation as follows: 0, no consolidation; 1, consolidation area < 1/3; 2, consolidation area 1/3 to 1/2; 3, consolidation area 1/2 to 2/3; 4, consolidation area > 2/3. The score increased with the severity of lung injury. The right lung tissue was used for evaluation of viral titer, transcriptome analysis, histopathological analysis and lipid mediator analysis.

### Influenza viral titer

The amount of influenza virus in infected mice was titrated by plaque assay. Right lung tissue was homogenized in Hank’s Balanced Salt Solution (Life Technologies Corporation) and diluted in Minimum Essential Media (MEM, GIBCO). An aliquot (0.1 mL) of lung homogenate solution was added to an MDCK culture plate (12-well-plate) and incubated for 1 h. The cells were cultured for 2 days in culture equipment at 5% CO_2_ and 37 °C. After the incubation period, the number of the viral plaques was counted.

### Histopathological analysis

The left lung tissue fixed by formaldehyde was embedded in paraffin block, sectioned, and stained hematoxylin and eosin for histopathological analysis. Hyperplasia/hypertrophy of bronchial mucosal epithelium, degeneration/necrosis of bronchial mucosal epithelium, infiltration of mononuclear cells and polymorphonuclear leukocytes in bronchus and alveolar septum, exudation of mononuclear cells and polymorphonuclear leukocytes atelectasis, edema and hemorrhage were scored. The score increased with the severity of findings.

### Wide-targeted lipid mediator analysis

#### Sample preparation

Lipid mediators in plasma samples were extracted by following a previously described solid-phase extraction (SPE) liquid chromatography (LC)–mass spectrometry (MS)/MS method^16^. In brief, 100 µL of plasma sample was mixed with 1 mL of methanol containing a mixture of internal standards (0.5 ng/μL each of tetranor-PGE metabolite (PGEM)-d6, thromboxane B2 (TXB2)-d4, PGE2-d4, PGD2-d4, leukotriene C4 (LTC4)-d5, LTB4-d4, 5-hydroxy-eicosatetraenoic acid (5-HETE)-d8, and 15-HETE-d8; 0.25 ng/μL of oleoylethanolamide-d4; and 10 ng/μL of AA-d8; all from Cayman Chemical, Ann Arbor, MI, USA) for 5 min at room temperature. After centrifugation, the supernatant was diluted with 4 mL of 0.1% formic acid in water and loaded onto the preconditioned SPE cartridge (STRATA-X, 10 mg/1 ml, Phenomenex, Torrance, CA, USA) with 1 mL each of 0.1% formic acid and 15% ethanol. The lipids were eluted with 250 μL of 0.1% formic acid in methanol. The extract was evaporated to dryness in vacuum evaporator and dissolved in 20 μL of methanol. Aliquots of 5 µL were injected into the LC/MS system.

Ice-cold methanol was added to the frozen lung sample (20 mg tissue/mL) and homogenized by Automill (TK-AM7-24, Tokken Inc.) for 30 s. Lung samples spiked with a mixture of internal standards were agitated at 4 °C for 60 min and then centrifuged at 13,000 rpm for 10 min at 4 °C (Model 3740, Micro Refrigerated Centrifuge, KUBOTA). After centrifugation, the supernatants were loaded onto SPE cartridges and separated as described for plasma samples.

#### LC–MS/MS analysis

Analyses were carried out with a Shimadzu Nexera X2-UHPLC system coupled to a Shimadzu LCMS-8050 triple quadrupole mass spectrometer. A reversed-phase column (Kinetex C8, 2.1 × 150 mm, 2.6 μm, Phenomenex) was used for chromatographic separation. Comprehensive analysis was performed by using LC–MS solution software and the LC–MS/MS Method Package for Lipid Mediators version 2 (Shimadzu). Metabolomics data were processed by using Excel software (Microsoft Corporation, Redmond, WA, USA). Missing values in the raw data were replaced by half of the minimum positive value, and these data were used for subsequent statistical analysis. The lipid mediator pathways were visualized by using VANTED software (https://www.cls.uni-konstanz.de/software/vanted/)^57^.

### Gene expression microarray analysis

Total RNA of lung tissue was isolated by using QIAzol (Qiagen, Valencia, CA, USA) with an RNAeasy kit (Qiagen) according to the manufacturer’s protocol. Microarray analysis was performed in accordance with the method reported by Akane et al.^58^. Gene expression analysis was performed by using a SurePrint G3 Mouse GE microarray (8 × 60 K v. 2.0) (Agilent Technologies, Santa Clara, CA, USA) The gene expression data were normalized and processed by GeneSpring GX (Agilent Technologies). The raw signal intensity was normalized by a 75% percentile shift. The probe ID was summarized in Entrez Gene ID, and the gene level intensity was used for analysis.

### Weighted gene co-expression network analysis

For all genes detected in gene expression microarray analysis, weighted gene co-expression network analysis (WGCNA) was conducted to identify gene modules associated with normal, IVI, and IVI plus MT conditions by R^59^ on the Garuda platform (www.garuda-alliance.org). The minimal gene number in the module was set as 50. The soft-thresholding power for scale-free topology was calculated; we obtained a power of soft threshold (β = 10) that satisfied more than 0.9 of the square of the model fitting index R (Fig. S4b).

### Cell type enrichment analysis

The cell type specificity of the gene set was analyzed by cell type enrichment (CTen) analysis (http://www.sbi.jp/influenza-x/cten)^60^. To analyze the module-specific cell type, the genes in each module were first individually input and the result obtained. We then input the gene sets that differed significantly between IVI and IVI + MT (*P* < 0.05 and FC > 1.5) in each module to analyze the effect of MT on cell type specificity. The CTen analysis for DEG was conducted using the gene set obtained from comparison between IVI and NI, and IVI + MT and IVI, respectively. To analyze the effect of IVI on NI, gene sets significantly increased or decreased by IVI relative to NI at criteria of *P* < 0.05 and log_2_FC > 3 or *P* < 0.05 and log_2_FC < –3 were used. To analyze the effect of maoto on IVI, gene sets significantly increased or decreased by MT relative to IVI at criteria of *P* < 0.05 and log_2_FC > 1 or *P* < 0.05 and log_2_FC < –1 were used. The enriched cell types were obtained at the criteria of enrichment score (-log_10_ Benjamini–Hochberg adjusted P) > 2.

### Pathway enrichment analysis

The pathway enrichment analysis for gene set in modules obtained by WGCNA and gene set obtained by DEG was conducted by using MetaCore software (ver. 6.11, build 41105, (Clarivate Analytics, USA, New York) (https://clarivate.com/products/metacore/). The gene sets significantly increased or decreased by IVI relative to NI at criteria of *P* < 0.05 and log_2_FC > 3 or log_2_FC < -3 were used for the analysis. The gene sets significantly increased or decreased by IVI + MT relative to IVI at criteria of P < 0.05 and FC > 1.5 or FC < -0.67 were used for WGCNA, and log_2_FC > 1 or log_2_FC < -1 were used for DEG. The enriched pathways at criteria of FDR < 0.05 and associated with viral infection, immune response and inflammation response were selected.

### Statistical analysis

The statistical significance of differences in rectal temperature, body weight, viral titer and lipid mediator were determined by Welch’s t-test with Bonferroni’s multiple comparisons. Differences in histopathological score were evaluated by Mann–Whitney U test with Bonferroni’s multiple comparisons, and those in survival and clinical signs were determined by Log-rank test. The data were analyzed and visualized by using GraphPad Prism7 software (GraphPad Software, San Diego, CA, USA). The significance level for each statistical analysis was set at *P* < 0.05.

The statistical significance of differences in lipid mediators was determined by Welch’s t-test with Bonferroni’s correction for multiple comparisons of the group using Excel software. The clustering analysis of lipid mediators was performed by using Metaboanalyst (https://www.metaboanalyst.ca), ^61^ and the data were visualized as a boxplot by ggplot2 in the R software package.

In transcriptome analysis, differential expression genes were analyzed by moderated *t* test with Benjamini Hochberg false discovery rate (FDR) multiple testing corrections using GeneSpring GX. The significance level was set at *p* < 0.05 and fold change criteria, which are mentioned in each analysis, were used as a threshold for enrichment analysis.

## Supplementary Information

Supplementary Information 1.

Supplementary Information 2.

## Data Availability

The metabolomics data measured by LC–MS/MS are shown in Figs. S2c and S3c. Experimental data are available from the authors. The microarray data have been recorded in GenBank (https://www.ncbi.nlm.nih.gov/genbank/) (accession number; GSE158270).
