# Supplementary material for: Effects of maoto (ma-huang-tang) on host lipid mediator and transcriptome signature in influenza virus infection
Source: Sci Rep. 2021 Feb 19;11:4232. doi: 10.1038/s41598-021-82707-1 (PMC7896050; doi:10.1038/s41598-021-82707-1)
Supplement: Supplementary file 1 — Supplementary Information 1. [file 41598_2021_82707_MOESM1_ESM.pdf]

Effects of maoto (ma-huang-tang) on host lipid mediator and transcriptome signature in influenza virus infection

Akinori Nishi[1], Noriko Kaifuchi[1], Chika Shimobori[1], Katsuya Ohbuchi[1], Seiichi Iizuka[1], Aiko Sugiyama[1], Keisuke Ogura[1], Masahiro Yamamoto[1], Haruo Kuroki[2], Shigeki Nabeshima[3], Ayako Yachie[4], Yukiko Matsuoka[4], Hiroaki Kitano[4]

1; Tsumura Kampo Research Laboratories, Tsumura & Co., Ibaraki, Japan.

2; Sotobo Children's Clinic, Medical Corporation Shigyo-no-kai, Isumi, Chiba, Japan

3; General Medicine, Fukuoka University Hospital, Fukuoka, Japan

4; The Systems Biology Institute, Shinagawa, Tokyo, Japan.

Correspondence to nishi_akinori@mail.tsumura.co.jp

Supplementary Methods

Materials

Maoto is an extracted mixture of Ephedrae Herba (32.3%), Armeniacae Semen (32.3%), Cinnamomi Cortex (25.8%), and Glycyrrhizae Radix (9.6%). Dry powdered extracts of maoto (supplied as TJ-27E, lot no. 341115500), industrially produced by spray-drying, were supplied by Tsumura & Co. (Tokyo, Japan). Maoto was dissolved in distilled water before administration to mice.

Virus culture

Mouse-adapted influenza virus [A/PR/8/34(H1N1)] (PR8) stocked at Nihon Bioresearch Inc. (Gifu, Japan), was used to infect mice. Madin–Darby canine kidney (MDCK) cells stocked at Nihon Bioresearch Inc. were cultured in DMEM with Fetal Bovine Serum (FBS, MultiSter Foetal Bovine Serum, Thermo Electron), antibiotics (Antibiotic-Antimycotic, Gibco) in culture equipment at 5% CO_2 and 37 °C. The influenza virus was cultured in MDCK cells, and PR8 collected from the culture medium was used for intranasal inoculation of mice.

Animals

Female BALB/c Cr Slc mice were purchased from Japan SLC, Inc. (Shizuoka, Japan) at 5 weeks of age, and used from 6 weeks of age after habituation. The mice were housed in cages with paper

chips (5 mice/cage) at a relative humidity of 40%–70%, a temperature of 19–24 ºC, and a 12-h light–dark cycle (6:00–18:00). The mice were permitted free access to food and water. All experiments were approved by the Laboratory Animal Committee of Tsumura & Co. and Nihon Bioresearch Inc. (Hashima, Gifu, Japan).

In vivo **influenza virus inoculation**

The influenza virus (1×10^5 PFU/0.05 mL/mouse) was administered by intranasal inoculation. Maoto (0.5 or 2 g/10mL/kg) was orally administrated for 5 days, starting 1 hour after inoculation. Mice with no inoculation mice were used as control mice against the infection, and infected mice that were treated distilled water were used as infected control mice. In total, there were four groups of mice: 1) no-inoculation (NI) group; 2) influenza virus inoculation (IVI) control group; 3) IVI with MT 0.5 g/10mL/kg treatment [IVI+MT(L)] group; and 4) IVI with MT 2 g/10mL/kg treatment [IVI+MT(H)] group. Daily body weight and clinical signs of each mouse were recorded. Clinical signs were divided into four categories, eye, fur, behavior and other (hypothermia, emaciation and respiratory failure), which were individually scored from 0 (normal) to 4 (death). The scores of the four categories were averaged per treatment group and the total clinical sign score was obtained. Rectal temperature was measured by using a thermometer (Physitemp, Model BAT-12, Physiotemp Instruments Inc.) by inserting the probe into the rectum of the mouse. Rectal temperature was

measured twice before inoculation and after inoculation, and then twice a day during the MT treatment period (before and 1 hour after MT treatment) and once a day after the MT treatment period.

Ten mice from each of the four mouse groups were used to record the survival period. Another 10 mice in each group were sacrificed at 5 days post inoculation (dpi) to collect tissue and plasma samples for analysis of viral titer, histopathological finding, transcriptome analysis, and lipid mediator analysis.

Tissue sampling

At 5 dpi, the mice were anesthetized with isoflurane (Mylan EPD G.K.), and the lung tissues and blood were obtained. Blood was collected from the inferior vena cava by heparinized syringe. The blood sample was centrifuged at 2150 × g at 4°C for 5 min to obtain the plasma, which was stored at –80ºC. Part of the left lung tissue was fixed by formaldehyde for histopathological analysis, and the other part was stored in –80ºC for subsequent lipid mediator analysis. The macroscopic findings of the lung tissue were scored by area of consolidation as follows: 0, no consolidation; 1, consolidation area <1/3; 2, consolidation area 1/3 to 1/2; 3, consolidation area 1/2 to 2/3; 4, consolidation area >2/3. The score increased with the severity of lung injury. Part of the right lung tissue was used for evaluation of viral titer; the other part was stored in RNAlater (Thermo Fisher) for subsequent

transcriptome analysis.

Influenza virus titer

The amount of influenza virus in infected mice was titrated by plaque assay. Right lung tissue was homogenized in Hank's Balanced Salt Solution (Life Technologies Corporation) and diluted in Minimum Essential Media (MEM, GIBCO). An aliquot (0.1 mL) of undiluted or diluted lung homogenate solution was added to an MDCK culture plate (12-well-plate) and incubated for 1 hour. The cells were overlaid with medium (1.5 mL) comprising MEM, 7.5% $NaHCO_3$, 200 mmol/L L-glutamine, 1% DEAE dextran, 15% glucose, 10% BSA, 2.5% trypsin, antibiotics and agarose, and cultured for 2 days in culture equipment at 5% CO_2 and 37 ^{o}C. After the incubation period, the cells were overlaid with medium containing neutral red and cultured for 1 day, and the number of the viral plaques was counted.

Histopathological analysis

The left lung tissue fixed by formaldehyde was embedded in paraffin block, sectioned, and stained with hematoxylin and eosin by a standard protocol for histopathological analysis.

Hyperplasia/hypertrophy of bronchial mucosal epithelium, degeneration/necrosis of bronchial mucosal epithelium, infiltration of mononuclear cells and polymorphonuclear leukocytes in

bronchus and alveolar septum, exudation of mononuclear cells and polymorphonuclear leukocytes atelectasis, edema and hemorrhage were scored. The score increased with the severity of findings.

Gene expression microarray analysis

Total RNA of lung tissue was isolated by using QIAzol (Qiagen, Valencia, CA, USA) with an RNAeasy kit (Qiagen) according to the manufacturer's protocol. Microarray analysis was performed in accordance with the method reported by Akane et al. In brief, gene expression analysis was performed by using a SurePrint G3 Mouse GE microarray (8 × 60K v. 2.0) (Agilent Technologies, Santa Clara, CA, USA) and Agilent's standard protocol (One-Color Microarray-Based Gene Expression Analysis Low Input Quick Amp Labeling, v. 6.9, December 2015) was referenced for sample preparation and array processing. As the control RNA, One-Color RNA Spike-In was prepared and mixed with total RNA. The mixture of total RNA was used for reverse transcription by Moloney Murine Leukemia Virus Reverse Transcriptase and cDNA including the T7 promoter primer was obtained. Using the cDNA as template, cRNA labeled by Cy3 was synthesized. The cRNA was hybridized to the microarray and scanned with a DNA microarray Scanner (Agilent). The data were obtained by using Agilent Feature Extraction Software (version 10.7.1.1) with default settings for all parameters. The gene expression data were normalized and processed by GeneSpring GX (Agilent Technologies). The raw signal intensity was normalized by a 75% percentile shift. The

probe ID was summarized in Entrez Gene ID, and the gene level intensity was used for analysis.
