# Supplementary material for: Effects of maoto (ma-huang-tang) on host lipid mediator and transcriptome signature in influenza virus infection
Source: Sci Rep. 2021 Feb 19;11:4232. doi: 10.1038/s41598-021-82707-1 (PMC7896050; doi:10.1038/s41598-021-82707-1)
Supplement: Supplementary file 2 — Supplementary Information 2. [file 41598_2021_82707_MOESM2_ESM.pdf]

Fig. S1

BW

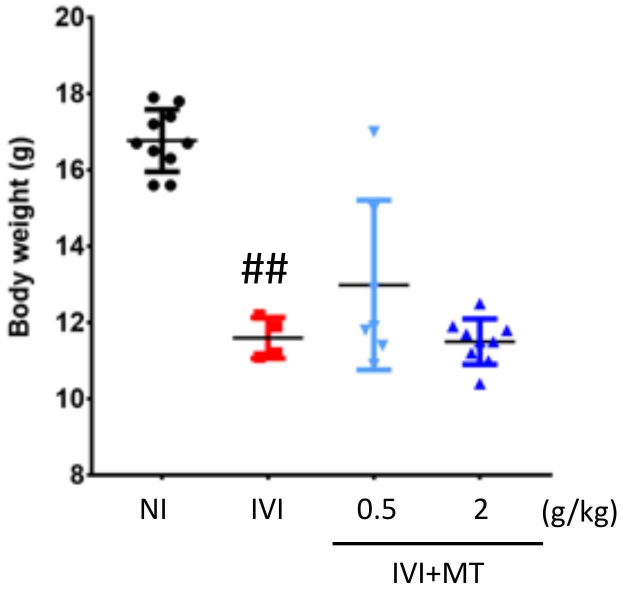

Fig. S2

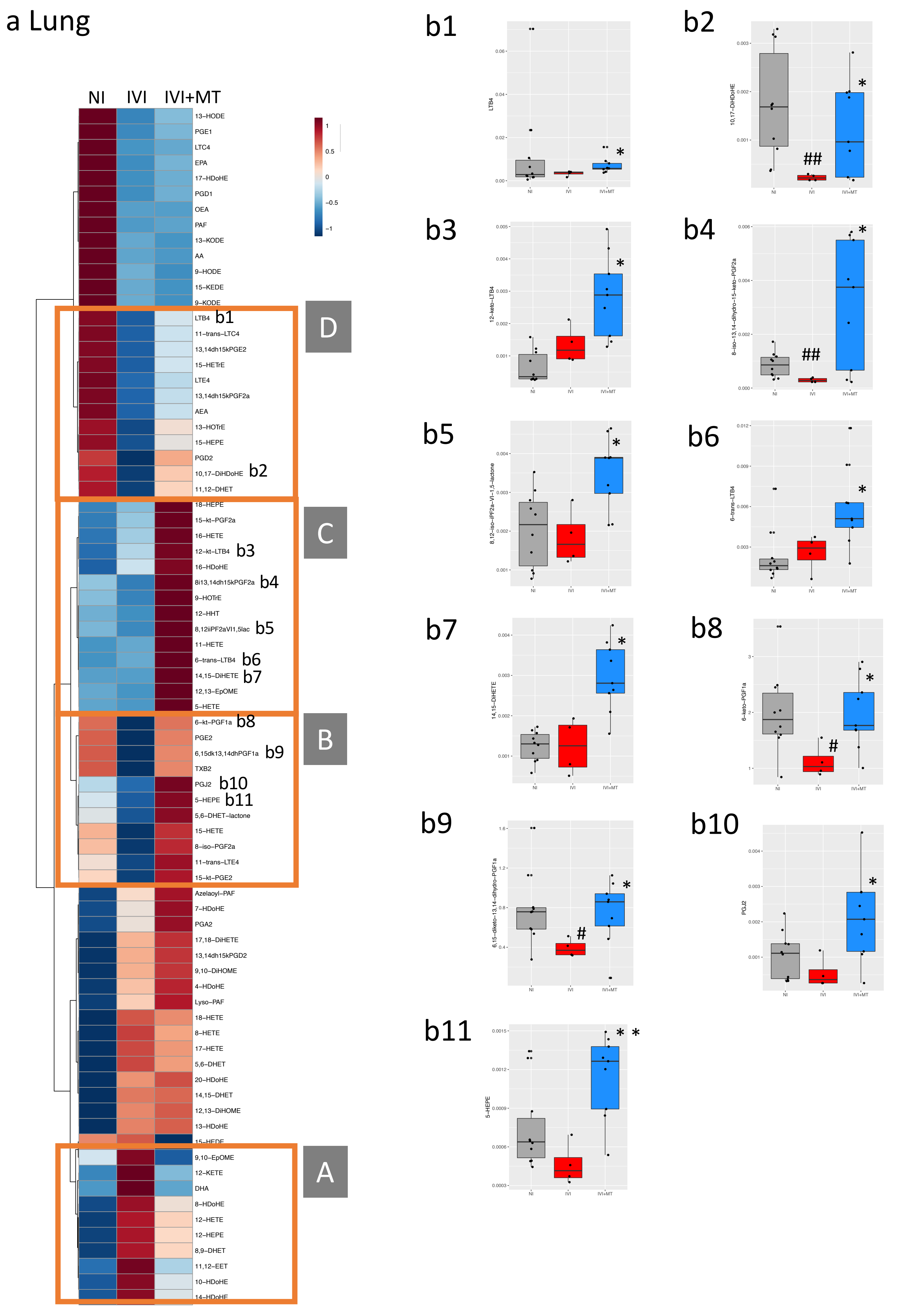

a Lung
NI
IVI
IVI+MT
13–HODE
PGE1
LTC4
EPA
17–HDoHE
PGD1
OEA
PAF
13–KODE
AA
9–HODE
15–KEDE
9–KODE
LTB4
11–trans–LTC4
13,14dh15kPGE2
15–HETrE
LTE4
13,14dh15kPGF2a
AEA
13–HOTrE
15–HEPE
PGD2
10,17–DiHDoHE
11,12–DHET
18–HEPE
15–kt–PGF2a
16–HETE
12–kt–LTB4
16–HDoHE
8i13,14dh15kPGF2a
9–HOTrE
12–HHT
8,12iiPF2aVI1,5lac
11–HETE
6–trans–LTB4
14,15–DiHETE
12,13–EpOME
5–HETE
6–kt–PGF1a
PGE2
6,15dk13,14dhPGF1a
TXB2
PGJ2
5–HEPE
5,6–DHET–lactone
15–HETE
8–iso–PGF2a
11–trans–LTE4
15–kt–PGE2
Azelaoyl–PAF
7–HDoHE
PGA2
17,18–DiHETE
13,14dh15kPGD2
9,10–DiHOME
4–HDoHE
Lyso–PAF
18–HETE
8–HETE
17–HETE
5,6–DHET
20–HDoHE
14,15–DHET
12,13–DiHOME
13–HDoHE
15–HEDE
9,10–EpOME
12–KETE
DHA
8–HDoHE
12–HETE
12–HEPE
8,9–DHET
11,12–EET
10–HDoHE
14–HDoHE
D
C
B
A
1
0.5
0
−0.5
−1
b1
b2
b3
b4
b5
b6
b7
b8
b9
b10
b11
LTB4
10,17–DiHDoHE
12–keto–LTB4
8–iso–13,14–dihydro–15–keto–PGF2a
8,12–iso–iPF2a–VI–1,5–lactone
6–trans–LTB4
14,15–DiHETE
6–keto–PGF1a
6,15–diketo–13,14–dihydro–PGF1a
PGJ2
5–HEPE
NI
IVI
IVI+MT
*
##
#
* *

Fig. S2

c lung

Metabolite	Category	Log2FC (IVI/NI)	Log2FC (IVI+MT/IVI)	P value (IVI vs NI)	P value (IVI+MT vs IVI)
9-HODE	LA	-0.59	-0.08	0.144	0.854
9-KODE	LA	-0.40	-0.04	0.178	0.911
13-HODE	LA	-0.39	0.07	0.232	0.845
13-KODE	LA	-0.21	-0.01	0.471	0.973
12,13-EpOME	LA	-0.07	0.91	0.853	0.028
9,10-EpOME	LA	0.16	-0.28	0.726	0.534
9,10-DiHOME	LA	0.50	0.13	0.303	0.735
12,13-DiHOME	LA	0.70	0.05	0.414	0.933
15-KEDE	EDA	-0.44	-0.04	0.119	0.894
15-HEDE	EDA	0.03	-0.32	0.954	0.553
PGE1	DGLA	-2.01	0.47	***0.002***	0.410
PGD1	DGLA	-1.48	0.23	***0.007***	0.680
15-HETrE	DGLA	-0.90	0.43	0.054	0.371
LTC4	AA	-9.12	5.09	0.079	0.212
PAF	AA	-5.96	1.20	***0.021***	0.169
11-trans-LTC4	AA	-5.32	4.00	0.109	0.348
LTE4	AA	-4.17	2.72	0.173	0.078
11-trans-LTE4	AA	-3.34	4.14	0.163	0.236
LTB4	AA	-1.86	1.06	0.227	***0.021***
8-iso-13,14-dihydro-15-keto-PGF2a	AA	-1.53	3.41	***0.004***	***0.006***
13,14-dihydro-15-keto-PGF2a	AA	-1.18	0.54	***0.009***	0.285
13,14-dihydro-15-keto-PGE2	AA	-1.06	0.51	***0.025***	0.312
6,15-diketo-13,14-dihydro-PGF1a	AA	-0.99	0.93	***0.009***	***0.012***
AA	AA	-0.94	-0.05	0.494	0.869
PGJ2	AA	-0.93	1.94	0.138	***0.007***
6-keto-PGF1a	AA	-0.82	0.81	***0.008***	***0.007***
TXB2	AA	-0.69	0.64	0.211	0.279
PGD2	AA	-0.65	0.53	0.280	0.390
5,6-DHET-lactone	AA	-0.53	0.98	0.217	0.026
15-keto-PGE2	AA	-0.43	0.65	0.434	0.275
15-HETE	AA	-0.39	0.50	0.330	0.220
PGE2	AA	-0.38	0.36	0.664	0.706
8-iso-PGF2a	AA	-0.22	0.30	0.629	0.469
8,12-iso-iPF2a-VI-1,5-lactone	AA	-0.15	0.93	0.676	***0.009***
12-HHT	AA	-0.11	0.92	0.803	0.062
11,12-DHET	AA	-0.04	0.03	0.901	0.924
5-HETE	AA	-0.03	0.64	0.958	0.086
11-HETE	AA	0.04	0.50	0.939	0.338
6-trans-LTB4	AA	0.14	1.21	0.804	***0.019***
15-keto-PGF2a	AA	0.15	0.45	0.816	0.451
14,15-DHET	AA	0.23	0.01	0.496	0.978
16-HETE	AA	0.25	0.56	0.498	0.065
8,9-DHET	AA	0.28	-0.10	0.460	0.708
11,12-EET	AA	0.32	-0.22	0.590	0.703
13,14-dihydro-15-keto-PGD2	AA	0.45	0.11	0.384	0.791
17-HETE	AA	0.81	-0.10	0.119	0.806
8-HETE	AA	1.02	-0.15	0.151	0.766
12-keto-LTB4	AA	1.02	1.08	0.096	***0.014***
PGA2	AA	1.11	0.59	0.067	0.233
18-HETE	AA	1.35	-0.10	***0.022***	0.700
5,6-DHET	AA	1.72	-0.18	***0.000***	0.441
12-HETE	AA	1.94	-0.46	0.107	0.487
12-KETE	AA	2.69	-1.84	0.194	0.250
15-HEPE	EPA	-2.29	1.54	***0.000***	0.063
5-HEPE	EPA	-0.69	1.31	0.055	***0.000***
EPA	EPA	-0.20	0.02	0.562	0.948
14,15-DiHETE	EPA	0.00	1.26	0.998	***0.006***
18-HEPE	EPA	0.35	1.07	0.508	0.031
17,18-DiHETE	EPA	0.56	0.15	0.033	0.396
12-HEPE	EPA	1.66	-0.46	0.167	0.543
10,17-DiHDoHE	DHA	-2.94	2.44	***0.002***	***0.015***
17-HDoHE	DHA	-0.19	0.02	0.713	0.976
16-HDoHE	DHA	0.16	0.22	0.551	0.474
13-HDoHE	DHA	0.26	0.02	0.596	0.971
DHA	DHA	0.47	-0.45	0.289	0.318
7-HDoHE	DHA	0.47	0.33	0.359	0.368
20-HDoHE	DHA	0.74	0.09	0.170	0.801
4-HDoHE	DHA	1.17	0.32	***0.019***	0.191
8-HDoHE	DHA	1.73	-0.57	0.141	0.431
14-HDoHE	DHA	1.79	-0.72	0.164	0.395
10-HDoHE	DHA	1.93	-0.78	0.140	0.352
13-HOTrE	ALA	-2.21	1.57	***0.003***	0.031
9-HOTrE	ALA	-0.20	0.91	0.716	0.047
OEA	EA	-0.78	-0.01	0.030	0.978
AEA	EA	-0.75	0.33	***0.001***	0.096
Lyso-PAF	-	0.82	0.32	***0.004***	0.038
Azelaoyl-PAF	-	1.37	0.54	0.029	0.222

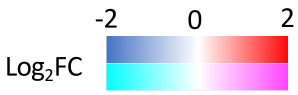

Fig. S3

a Plasma

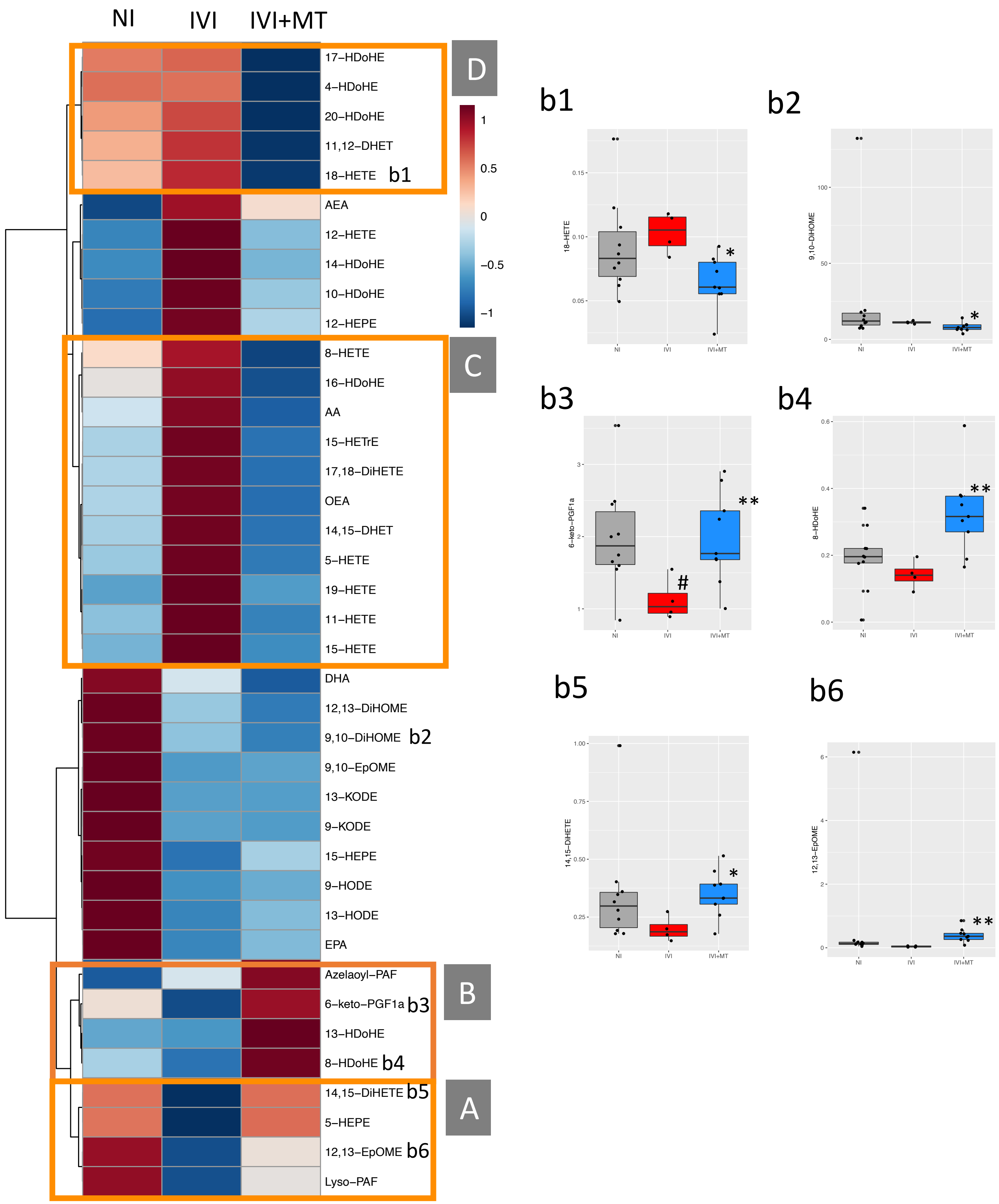

Fig. S3

c plasma

Metabolite	Category	Log_2FC (IVI/NI)	Log_2FC (IVI+MT/IVI)	P value (IVIvsNI)	P value (IVI+MT vs IVI)
13-KODE	LA	-4.71	-0.40	0.332	0.606
9-KODE	LA	-4.55	-0.80	0.339	0.222
12,13-EpOME	LA	-4.20	3.32	0.279	***0.001***
9,10-EpOME	LA	-2.65	0.16	0.322	0.567
9-HODE	LA	-2.17	0.34	0.364	0.593
13-HODE	LA	-1.87	0.50	0.310	0.426
12,13-DiHOME	LA	-1.11	-0.58	0.322	0.196
9,10-DiHOME	LA	-1.12	-0.47	0.304	***0.017***
15-HETrE	DGLA	0.62	-0.96	0.457	0.279
6-keto-PGF1a	AA	-1.58	2.25	***0.022***	***0.002***
11,12-DHET	AA	0.10	-0.44	0.875	0.291
8-HETE	AA	0.25	-0.70	0.752	0.365
AA	AA	0.26	-0.45	0.265	0.099
14,15-DHET	AA	0.54	-0.80	0.132	0.043
5-HETE	AA	0.78	-1.12	0.408	0.294
15-HETE	AA	0.94	-1.11	0.240	0.147
11-HETE	AA	1.07	-1.45	0.270	0.170
19-HETE	AA	1.15	-1.19	0.070	0.067
12-HETE	AA	4.76	-2.40	0.102	0.144
18-HETE	AA	0.16	-0.67	0.445	***0.007***
14,15-DiHETE	EPA	-0.81	0.82	0.089	***0.005***
EPA	EPA	-1.62	0.38	***0.000***	0.136
15-HEPE	EPA	-0.77	0.25	0.136	0.625
5-HEPE	EPA	-0.30	0.30	0.555	0.330
17,18-DiHETE	EPA	0.39	-0.60	0.197	0.078
12-HEPE	EPA	3.00	-1.38	0.069	0.144
8-HDoHE	DHA	-0.44	1.20	0.197	***0.002***
DHA	DHA	-0.59	-0.66	0.059	0.137
13-HDoHE	DHA	-0.02	0.38	0.967	0.299
4-HDoHE	DHA	0.00	-1.00	0.996	0.052
17-HDoHE	DHA	0.03	-0.73	0.959	0.277
20-HDoHE	DHA	0.15	-1.41	0.835	0.060
16-HDoHE	DHA	0.26	-0.56	0.630	0.231
10-HDoHE	DHA	1.42	-0.98	0.168	0.252
14-HDoHE	DHA	3.90	-2.47	0.090	0.117
OEA	EA	0.56	-0.88	0.081	0.032
AEA	EA	0.66	-0.25	0.088	0.369
Lyso-PAF	-	-0.21	0.11	0.617	0.442
Azelaoyl-PAF	-	0.20	0.23	0.793	0.662

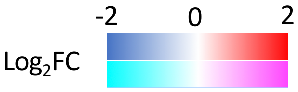

Fig. S4

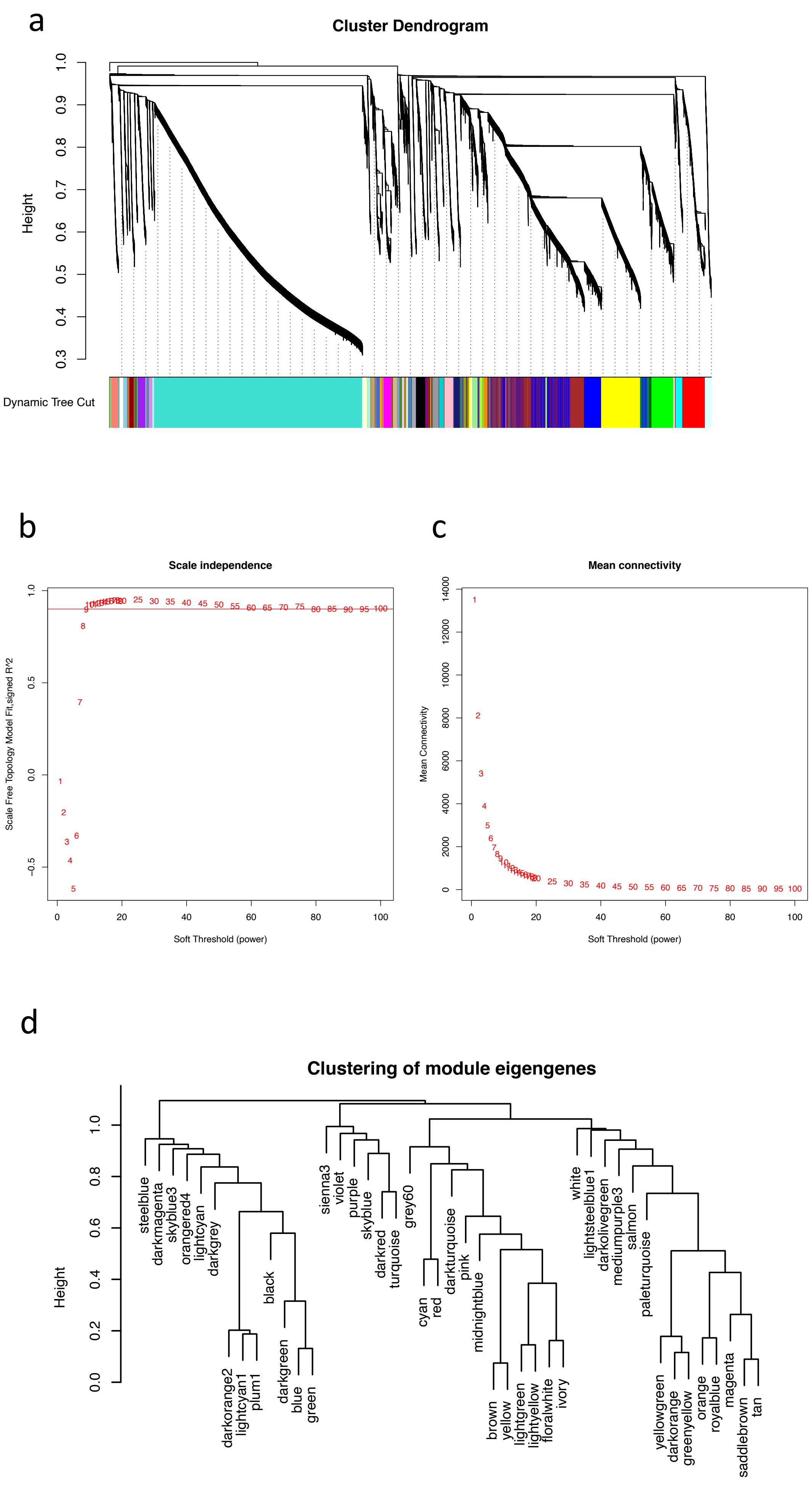
a
Cluster Dendrogram
Height
1.0
0.9
0.8
0.7
0.6
0.5
0.4
0.3
Dynamic Tree Cut
b
Scale independence
Scale Free Topology Model Fit,signed R^2
Soft Threshold (power)
c
Mean connectivity
Mean Connectivity
Soft Threshold (power)
d
Clustering of module eigengenes
Height
steelblue
darkmagenta
skyblue3
orangered4
lightcyan
darkgrey
darkorange2
lightcyan1
plum1
black
darkgreen
blue
green
sienna3
violet
purple
skyblue
darkred
turquoise
grey60
cyan
red
darkturquoise
pink
midnightblue
brown
yellow
lightgreen
lightyellow
floralwhite
ivory
white
lightsteelblue1
darkolivegreen
mediumpurple3
salmon
paleturquoise
yellowgreen
darkorange
greenyellow
orange
royalblue
magenta
saddlebrown
tan

Fig. S4

e

module	correlation			p value		
	NI	IVI	IVI+MT	NI	IVI	IVI+MT
green	-0.897	0.088	0.843	***6.6E-09***	6.9E-01	***4.4E-07***
darkorange2	-0.291	-0.667	0.814	1.8E-01	***5.1E-04***	***2.3E-06***
darkgreen	-0.574	-0.255	0.781	***4.2E-03***	2.4E-01	***1.1E-05***
lightcyan1	-0.228	-0.679	0.759	2.9E-01	***3.7E-04***	***2.6E-05***
blue	-0.995	0.455	0.657	***1.3E-22***	***2.9E-02***	***6.6E-04***
plum1	0.182	-0.892	0.508	4.1E-01	***1.1E-08***	***1.3E-02***
darkorange	-0.627	0.205	0.477	***1.4E-03***	3.5E-01	***2.1E-02***
brown	-0.898	0.693	0.373	***6.4E-09***	***2.5E-04***	7.9E-02
skyblue3	-0.191	-0.097	0.269	3.8E-01	6.6E-01	2.1E-01
darkmagenta	-0.189	-0.098	0.268	3.9E-01	6.6E-01	2.2E-01
steelblue	-0.190	-0.096	0.267	3.9E-01	6.6E-01	2.2E-01
black	-0.189	-0.095	0.266	3.9E-01	6.6E-01	2.2E-01
darkgrey	-0.187	-0.097	0.265	3.9E-01	6.6E-01	2.2E-01
lightcyan	-0.186	-0.099	0.265	4.0E-01	6.5E-01	2.2E-01
paleturquoise	-0.184	-0.099	0.264	4.0E-01	6.5E-01	2.2E-01
orangered4	-0.170	-0.109	0.257	4.4E-01	6.2E-01	2.4E-01
grey60	-0.181	-0.093	0.256	4.1E-01	6.7E-01	2.4E-01
greenyellow	-0.574	0.529	0.172	***4.1E-03***	***9.4E-03***	4.3E-01
yellowgreen	-0.195	0.092	0.126	3.7E-01	6.8E-01	5.7E-01
yellow	-0.690	0.889	0.010	***2.7E-04***	***1.4E-08***	9.6E-01
orange	-0.039	0.112	-0.047	8.6E-01	6.1E-01	8.3E-01
royalblue	0.016	0.094	-0.090	9.4E-01	6.7E-01	6.8E-01
saddlebrown	0.470	-0.468	-0.114	***2.4E-02***	***2.4E-02***	6.1E-01
magenta	0.132	-0.009	-0.127	5.5E-01	9.7E-01	5.7E-01
red	-0.034	0.217	-0.134	8.8E-01	3.2E-01	5.4E-01
darkred	0.232	-0.089	-0.167	2.9E-01	6.9E-01	4.5E-01
mediumpurple3	0.239	-0.098	-0.167	2.7E-01	6.6E-01	4.5E-01
white	0.243	-0.096	-0.172	2.6E-01	6.6E-01	4.3E-01
lightsteelblue1	0.243	-0.096	-0.172	2.6E-01	6.6E-01	4.3E-01
darkturquoise	-0.186	0.464	-0.172	4.0E-01	***2.6E-02***	4.3E-01
pink	-0.181	0.461	-0.174	4.1E-01	***2.7E-02***	4.3E-01
violet	0.238	-0.082	-0.178	2.7E-01	7.1E-01	4.2E-01
skyblue	0.239	-0.083	-0.178	2.7E-01	7.1E-01	4.2E-01
sienna3	0.242	-0.086	-0.179	2.7E-01	7.0E-01	4.1E-01
salmon	0.212	-0.045	-0.180	3.3E-01	8.4E-01	4.1E-01
midnightblue	-0.191	0.487	-0.184	3.8E-01	***1.8E-02***	4.0E-01
purple	0.245	-0.082	-0.185	2.6E-01	7.1E-01	4.0E-01
tan	0.290	-0.140	-0.186	1.8E-01	5.3E-01	3.9E-01
cyan	-0.180	0.476	-0.186	4.1E-01	***2.2E-02***	3.9E-01
darkolivegreen	0.241	-0.076	-0.186	2.7E-01	7.3E-01	3.9E-01
floralwhite	-0.033	0.603	-0.435	8.8E-01	***2.3E-03***	***3.8E-02***
ivory	-0.117	0.820	-0.519	6.0E-01	***1.6E-06***	***1.1E-02***
lightgreen	-0.118	0.835	-0.529	5.9E-01	***7.2E-07***	***9.5E-03***
turquoise	0.992	-0.491	-0.626	***3.2E-20***	***1.7E-02***	***1.4E-03***
lightyellow	0.306	0.671	-0.833	1.5E-01	***4.5E-04***	***8.2E-07***

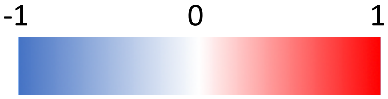

f

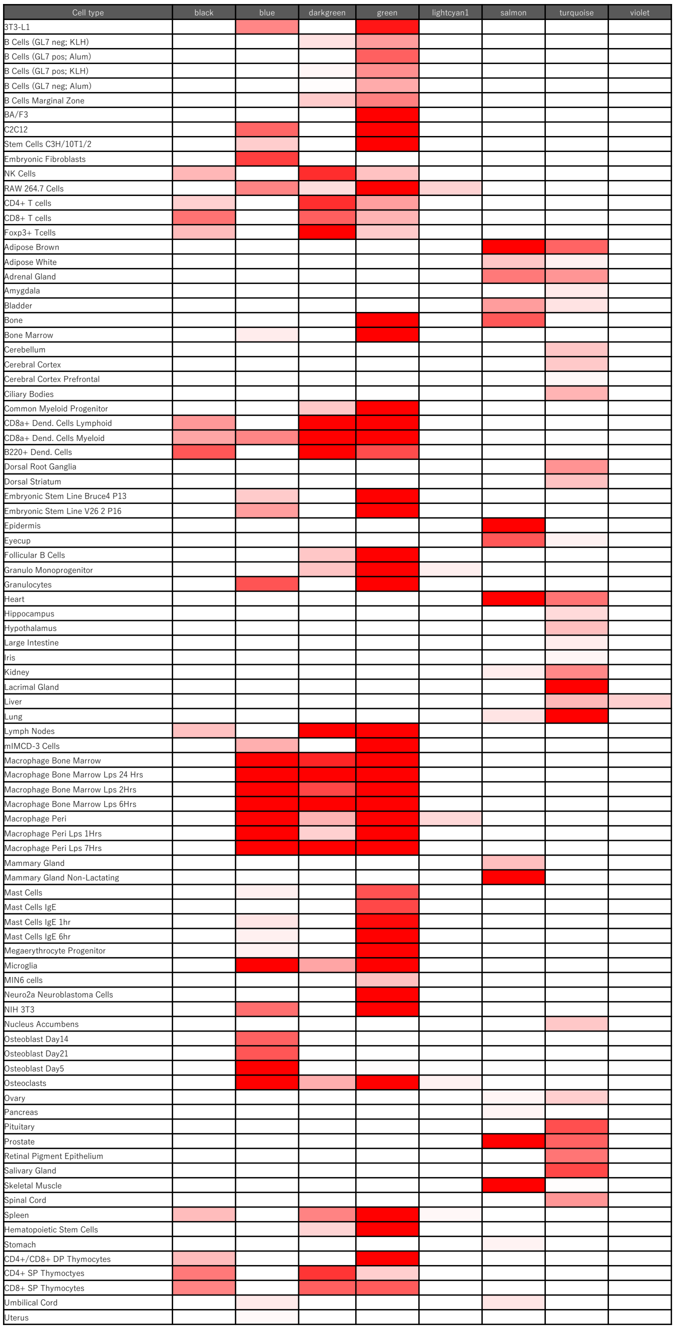

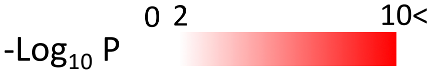

g

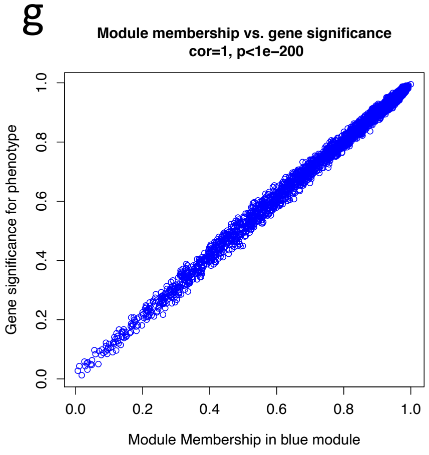

h

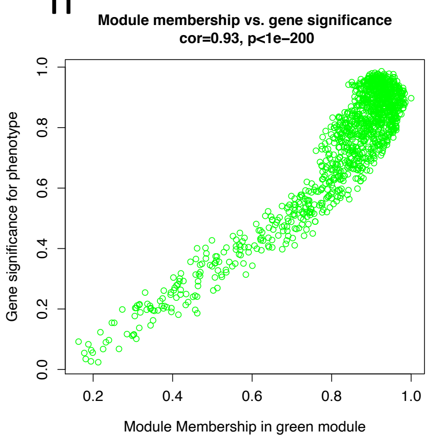

i

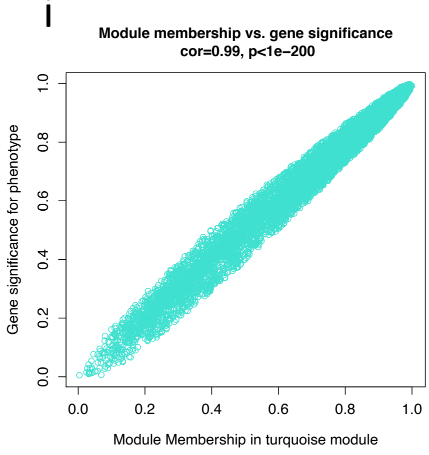

Fig. S4

j CTen analysis for WGCNA modules

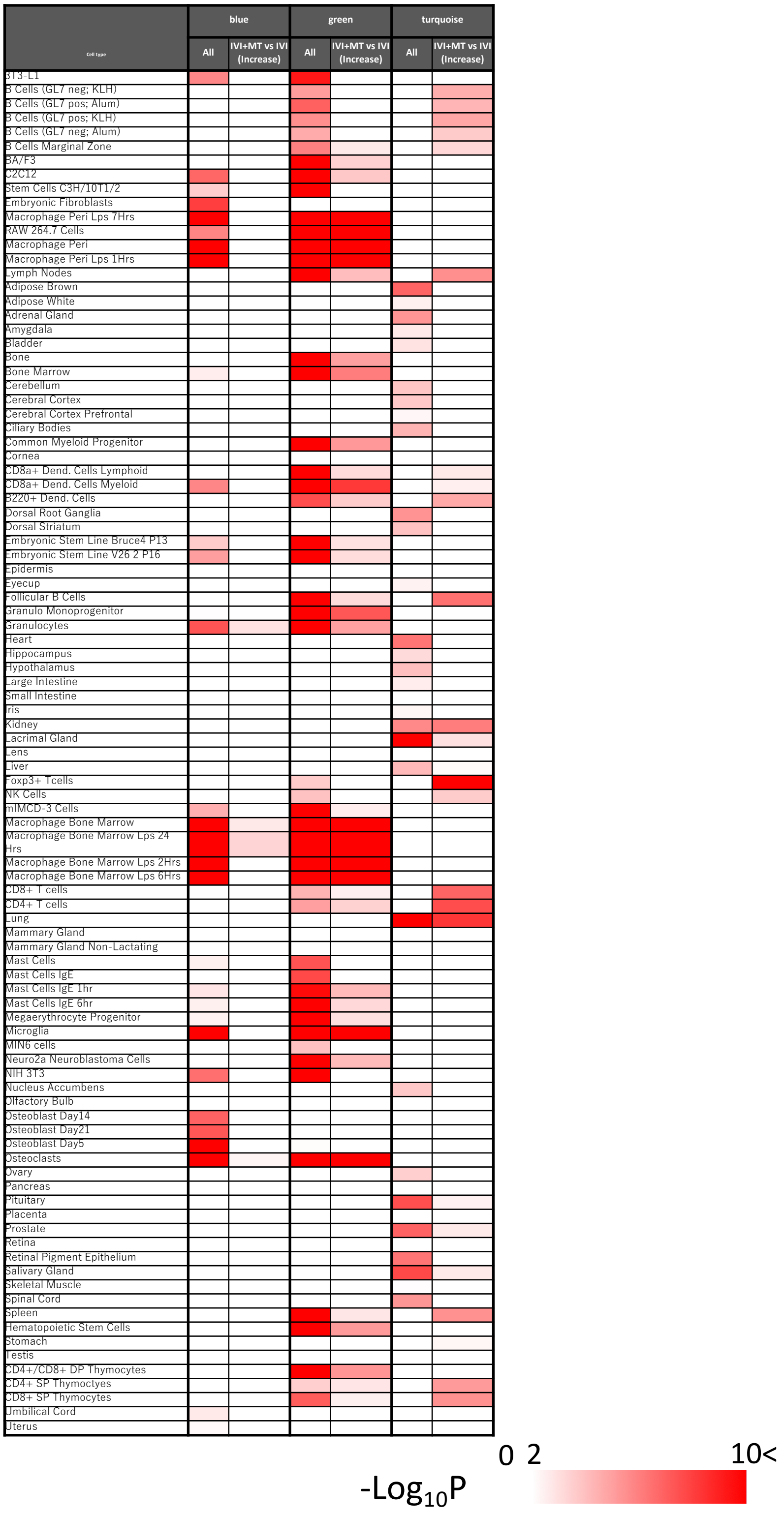

Fig. S5

Pathway enrichment analysis of differentially expressed gene between IVI and NI

a Increase

Maps	Total	IVI vs NI			
		p-value	FDR	In Data	Network Objects from Active Data
Immune response_IFN-alpha/beta signaling via JAK/STAT	64	1.371E-14	1.243E-11	16	p21, IFN-beta, Mx1, ISG54, IRF7, RSAD2, CCL2, IL1RN, SOCS3, OAS1, MIG, IFN-alpha, USP18, IP10, GBP4, ISG15
Immune response_IL-1 signaling pathway	82	1.938E-09	5.859E-07	13	FGF2, AP-1, IFN-beta, SPHK1, MMP-9, Collagen II, GRO-1, IL-6, CCL2, EGR1, IP10, CCL7, TNF-alpha
Release of pro-inflammatory factors and proteases by alveolar macrophages in asthma	44	3.860E-09	8.753E-07	10	MMP-9, GRO-2, GRO-1, IL-6, CCL2, MIG, TIMP1, Stromelysin-1, IP10, TNF-alpha
Immune response_IL-10 signaling pathway	62	1.030E-08	1.856E-06	11	ALOX5AP, MMP-9, IL-6, Heme oxygenase 1, Fc gamma RI, Fc gamma RII beta, SOCS3, G-CSF, TIMP1, CD14, TNF-alpha
NF-kB-, AP-1- and MAPKs-mediated proinflammatory cytokine production by eosinophils in asthma	43	5.147E-08	5.835E-06	9	AP-1, Myeloblastin, GRO-1, IL-6, CCL2, IL-25 (IL-17E), MIP-1-beta, Cathepsin G, TNF-alpha
Signal transduction_PDGF signaling via MAPK cascades	65	2.009E-07	1.551E-05	10	p21, AP-1, SPHK1, Tissue factor, MMP-9, Fra-1, IL-6, EGR1, Stromelysin-1, c-Myc
Immune response_IFN-gamma in macrophages activation	50	2.052E-07	1.551E-05	9	IL-6, PAI2, CCL2, Thrombospondin 1, Fc gamma RI, MIG, Factor B, IP10, TNF-alpha
Neutrophil chemotaxis in asthma	38	2.774E-07	1.936E-05	8	GRO-3, BDKRB1, GRO-2, GRO-1, CCL2, PLGF, PTAFR, CCL7
IL-1 beta- and Endothelin-1-induced fibroblast/ myofibroblast migration and extracellular matrix production in asthmatic airways	40	4.231E-07	2.711E-05	8	AP-1, PAI1, CCL2, Thrombospondin 1, TIMP1, Stromelysin-1, HAS2, Versican
IL-17 and IL-17F-induced inflammatory signaling in normal and asthmatic airway epithelium	28	4.484E-07	2.711E-05	7	GRO-1, IL-6, IL-19, G-CSF, IL-11, IP10, GCP2

Threshold	
Log_2FC	3
P-value	0.05

b Decrease

Maps	Total	IVI vs NI			
		p-value	FDR	In Data	Network Objects from Active Data
Immune response_IFN-alpha/beta signaling via JAK/STAT	64	4.754E-11	5.248E-08	16	p21, Mx1, IRF7, CCL2, OAS1, MIG, IFN-alpha, IFN-beta, ISG54, RSAD2, IL1RN, SOCS3, USP18, IP10, GBP4, ISG15
Neutrophil chemotaxis in asthma	38	6.688E-10	3.692E-07	12	BDKRB1, GRO-2, CCR3, CCL2, PLGF, IL8RA, PTAFR, GRO-3, C5a, GRO-1, Tissue kallikreins, CCL7
NF-kB-, AP-1- and MAPKs-mediated proinflammatory cytokine production by eosinophils in asthma	43	4.115E-08	1.514E-05	11	IL-6, CCL2, MIP-1-beta, Cathepsin G, AP-1, Myeloblastin, C5a, GRO-1, IL-25 (IL-17E), Chymase, TNF-alpha
Release of pro-inflammatory factors and proteases by alveolar macrophages in asthma	44	5.730E-07	9.739E-05	10	GRO-2, IL-6, CCL2, MIG, Stromelysin-1, MMP-9, GRO-1, TIMP1, IP10, TNF-alpha
Immune response_IL-1 signaling pathway	82	9.910E-07	1.368E-04	13	SPHK1, IL-6, CCL2, EGR1, FGF2, AP-1, IFN-beta, MMP-9, Collagen II, GRO-1, IP10, CCL7, TNF-alpha
Neutrophil-derived granule proteins and cytokines in asthma	49	1.658E-06	2.034E-04	10	Oncostatin M, IL8RA, Fc epsilon RI gamma, PTAFR, MMP-9, C5a, MMP-8, IP10, PERM, TNF-alpha
Immune response_IL-10 signaling pathway	62	2.169E-06	2.177E-04	11	ALOX5AP, IL-6, Heme oxygenase 1, Fc gamma RI, Fc gamma RII beta, G-CSF, MMP-9, SOCS3, TIMP1, CD14, TNF-alpha
Eosinophil chemotaxis in asthma	64	3.005E-06	2.765E-04	11	CCR3, IL8RA, CCR6, CCL8, PTAFR, C5a, C3aR, IP10, CCL13, Eotaxin-2, CCL7
Immune response_Alternative complement pathway	53	3.531E-06	2.999E-04	10	C5, PTX3, C6, Factor Ba, C5a, C5b, Factor Bb, C3aR, Factor B, CD21
Basophil migration in asthma	55	5.019E-06	3.958E-04	10	CCR3, CCL2, IL8RA, CCL8, P-selectin, MMP-9, C5a, CCL13, Eotaxin-2, CCL7
Immune response_IL-17 signaling pathways	60	1.131E-05	7.077E-04	10	Mucin 5B, IL-6, CCL2, G-CSF, Stromelysin-1, NGAL, MMP-9, GRO-1, CCL7, GCP2
Common mechanisms of Th17 cell migration	48	1.154E-05	7.077E-04	9	CCL2, MIP-1-beta, CCR6, MIG, CCL8, P-selectin, Collagen II, IP10, CCL7

Threshold	
Log_2FC	-3
P-value	0.05

Fig. S5

c

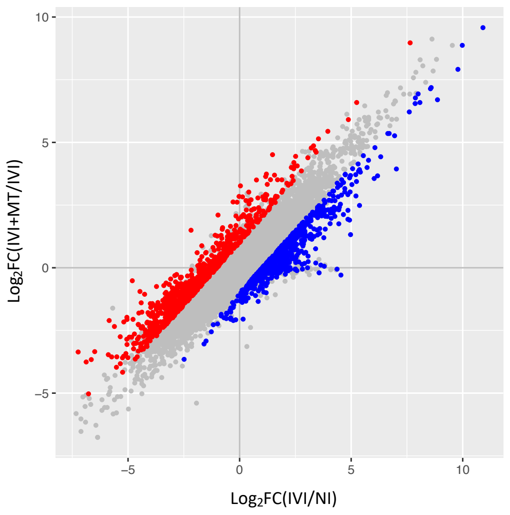

d

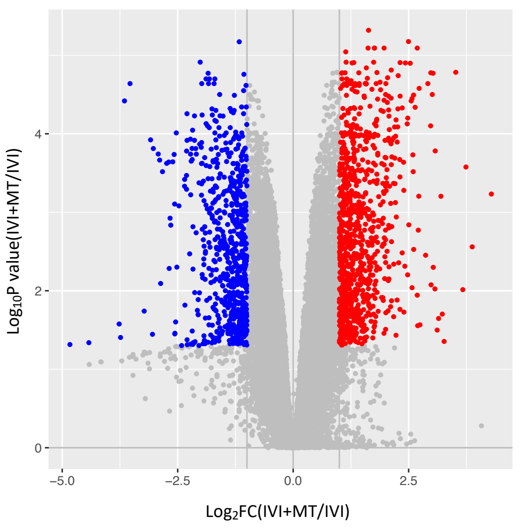

Pathway enrichment analysis of differentially expressed gene between IVI+MT and IVI

e Increase

	IVI+MT vs IVI				
**Maps**	**Total**	**p-value**	**FDR**	**In Data**	**Network Objects from Active Data**
Immune response_T cell subsets: cell surface markers	52	1.153E-13	9.121E-11	14	CD161, CD8, CCR3, ST2(L), CCR6, Klrb1c, IL-2R alpha chain, IL7RA, CCR4, CCR5, IL17BR, CXCR3, GITR, IL27RA
Immune response_T cell co-signaling receptors, schema	55	2.545E-08	1.006E-05	10	CD27(TNFRSF7), TIM-1, ICOS, TIM-4, LAIR1, PD-1, PD-L2, CD2, GITRL, GITR
Common mechanisms of Th17 cell migration	48	9.792E-08	2.582E-05	9	CD43, CD161, MIP-1-beta, CCR6, CXCR6, CCR4, CCR5, CCR2, CXCR3
Role of CD8+ Tc1 cells in COPD	44	6.614E-07	1.046E-04	8	MMP-12, CD8, T-bet, CXCR6, Granzyme B, CX3CR1, CCR5, CXCR3
Differentiation of Th2 cells in asthma	45	1.003E-05	1.219E-03	7	Histamine H1 receptor, CCR6, CCR4, CCR5, CCR2, CXCR3, PI3K reg class IA
Eosinophil chemotaxis in asthma	64	1.233E-05	1.219E-03	8	CCR3, IL8RA, CCR6, CysLT1 receptor, C5a, C3aR, IL-5, CXCR3
Eosinophil survival in asthma	67	1.739E-05	1.374E-03	8	PGE2R2, CCR3, IL-9, ST2(L), CysLT1 receptor, IL-5, iNOS, Apo-2L(TNFSF10)
Immune response_Lectin induced complement pathway	50	2.053E-05	1.374E-03	7	C5, C6, Factor I, C5a, C5b, C3aR, CD21
Immune response_Classical complement pathway	53	3.034E-05	1.500E-03	7	C5, C6, Factor I, C5a, C5b, C3aR, CD21
Immune response_Alternative complement pathway	53	3.034E-05	1.500E-03	7	C5, C6, Factor I, C5a, C5b, C3aR, CD21
Proinflammatory cytokine production by Th17 cells in asthma	53	3.034E-05	1.500E-03	7	C5, IL-9, CCR6, CCR4, C5a, C3aR, IL-5
Mast cell migration in asthma	73	3.283E-05	1.528E-03	8	CCR3, IL8RA, CXCR6, CCR4, C5a, C3aR, CCR2, CXCR3

Threshold	
Log_2FC	1
P-value	0.05

f Decrease

	IVI+MT vs IVI				
**Maps**	**Total**	**p-value**	**FDR**	**In Data**	**Network Objects from Active Data**
Immune response_IL-17 signaling pathways	60	1.020E-06	9.454E-04	8	GRO-1, IL-6, TRAF6, I-kB, G-CSF, Stromelysin-1, IL-17F, c-Fos
Transcription_HIF-1 targets	95	3.966E-06	1.434E-03	9	p21, FGF2, MCT4, Adrenomedullin, DEC1 (Stra13), REDD1, PLAUR (uPAR), NUR77, IBP3
NF-kB-, AP-1- and MAPKs-mediated proinflammatory cytokine production by eosinophils in asthma	43	1.846E-05	2.834E-03	6	Myeloblastin, PRG2, GRO-1, IL-6, Leukocyte elastase, IL-17F
Role of Th17 cells in asthma	28	2.751E-05	2.834E-03	5	GRO-3, GRO-2, GRO-1, IL-17F, IL-22
IL-17 and IL-17F-induced inflammatory signaling in normal and asthmatic airway epithelium	28	2.751E-05	2.834E-03	5	GRO-1, IL-6, I-kB, G-CSF, IL-17F
Development_Transcription regulation of granulocyte development	32	5.405E-05	4.324E-03	5	Myeloblastin, Leukocyte elastase, G-CSF, PKC, PERM
Oxidative stress_ROS-induced cellular signaling	108	8.267E-05	5.329E-03	8	p21, TXNIP (VDUP1), IL-6, Adrenomedullin, NFKBIA, GADD45 alpha, EGR1, PKC
Skeletal muscle atrophy in COPD	57	9.455E-05	5.329E-03	6	REDD1, NFKBIA, I-kB, FOXO3A, MyHC, MYOG
Immune response_IL-1 signaling pathway	82	9.500E-05	5.329E-03	7	FGF2, IFN-beta, GRO-1, IL-6, TRAF6, I-kB, EGR1
Role of alveolar cell senescence in COPD	37	1.110E-04	5.329E-03	5	p21, IL-6, I-kB, FOXO3A, IBP3
Neutrophil chemotaxis in asthma	38	1.265E-04	5.329E-03	5	GRO-3, GRO-2, GRO-1, Substance P extracellular region, Tissue kallikreins
Transcription_Role of AP-1 in regulation of cellular metabolism	38	1.265E-04	5.329E-03	5	p21, IFN-beta, PLAUR (uPAR), HBB, c-Fos
TNF-alpha-induced inflammatory signaling in normal and asthmatic airway epithelium	38	1.265E-04	5.329E-03	5	GRO-1, IL-6, NFKBIA, I-kB, c-Fos

Threshold	
Log_2FC	-1
P-value	0.05

Fig. S5

g Cell type enrichment analysis

Cell Type	IVI/NI		IVI+MT/IVI	
	Increase	Decrease	Increase	Decrease
Macrophage Peri Lps 7Hrs				
Macrophage Bone Marrow Lps 6Hrs				
Macrophage Bone Marrow Lps 24 Hrs				
Microglia				
Macrophage Bone Marrow Lps 2Hrs				
Macrophage Peri Lps 1Hrs				
Granulocytes				
Osteoclasts				
Macrophage Bone Marrow				
Osteoblast Day21				
Bone				
Bone Marrow				
3T3-L1				
RAW 264.7 Cells				
Macrophage Peri				
Osteoblast Day14				
Osteoblast Day5				
CD8a+ Dend. Cells Myeloid				
Mast Cells IgE 1hr				
Embryonic Fibroblasts				
Lymph Nodes				
Umbilical Cord				
Epidermis				
Granulo Monoprogenitor				
Mast Cells IgE				
mIMCD-3 Cells				
Mast Cells IgE 6hr				
Placenta				
Uterus				
Spleen				
C2C12				
Stem Cells C3H/10T1/2				
Mast Cells				
Mammary Gland				
Common Myeloid Progenitor				
Cornea				
Hematopoietic Stem Cells				
BA/F3				
CD8a+ Dend. Cells Lymphoid				
Small Intestine				
Large Intestine				
NIH 3T3				
Stomach				
B220+ Dend. Cells				
Adipose White				
Mammary Gland Non-Lactating				
Lens				
Bladder				
Salivary Gland				
CD4+/CD8+ DP Thymocytes				
NK Cells				
Liver				
Ovary				
Foxp3+ Tcells				
Ciliary Bodies				
Adipose Brown				
Iris				
Prostate				
Adrenal Gland				
Lung				
Pancreas				
Dorsal Root Ganglia				
Hippocampus				
Pituitary				
CD4+ T cells				
B Cells (GL7 pos; KLH)				
CD4+ SP Thymoctyes				
Follicular B Cells				
B Cells Marginal Zone				
Lacrimal Gland				
B Cells (GL7 neg; Alum)				
B Cells (GL7 pos; Alum)				
Kidney				
CD8+ T cells				
B Cells (GL7 neg; KLH)				
Olfactory Bulb				
MIN6 cells				
Cerebellum				
Cerebral Cortex				
Spinal Cord				
Retinal Pigment Epithelium				
Skeletal Muscle				
Retina				
Cerebral Cortex Prefrontal				
CD8+ SP Thymocytes				
Amygdala				
Nucleus Accumbens				
Hypothalamus				
Dorsal Striatum				

$-Log_{10}P$ 0 2 10<

Table S1

Histopathological score

Pneumonia score	IVI	IVI+MT (0.5 g/kg)	IVI+MT (2 g/kg)
Hyperplasia/hypertrophy, bronchial mucosal epithelium	0.75 ± 0.50	2.00 ± 1.15	1.56 ± 0.73
Degeneration/necrosis, bronchial mucosal epithelium	4.00 ± 0.00	1.86 ± 0.9**	2.78 ± 0.67*
Infiltration of mononuclear cells and polymorphonuclear leukocytes, bronchus	2.25 ± 0.50	2.29 ± 0.76	1.89 ± 0.60
Atelectasis, alveoli	0.25 ± 0.50	0.86 ± 1.07	0.22 ± 0.44
Edema, alveoli	0.75 ± 0.50	0.43 ± 0.53	0.44 ± 0.53
Hemorrhage, alveoli	0.50 ± 0.58	0.71 ± 0.76	0.89 ± 0.93
Infiltration of mononuclear cells and polymorphonuclear leukocytes, alveolar septum	2.75 ± 0.50	2.57 ± 0.79	2.78 ± 0.44
Exudation of mononuclear cells and polymorphonuclear leukocytes, alveoli	1.50 ± 0.58	2.14 ± 1.21	2.22 ± 0.44

Mean ± SD

Table S2

List of lipid mediators

Abbreviation	Name
4-HDoHE	4-hydroxy-docosahexaenoic acid
5-HEPE	5-hydroxy-eicosapentaenoic acid
5-HETE	5-hydroxy-eicosatetraenoic acid
5-HpEPE	5-hydroperoxy-eicosapentaenoic acid
5-HpETE	5-hydroperoxy-eicosatetraenoic acid
5-iPF2a-VI	5-iso-prostaglandin F2alpha-VI
5-KETE	5-keto-eicosatetraenoic acid
5,15-DiHETE	5,15-dihydroxy-eicosatetraenoic acid
5,6-DHET	5,6-dihydroxy-eicosatrienoic acid
5,6-DHET-lactone	5,6-dihydroxy-eicosatrienoic acid lactone
5,6-DiHETE	5,6-dihydroxy-eicosatetraenoic acid
5,6-EET	5,6-epoxy-eicosatrienoic acid
5,6-EET-EA	5,6-epoxy-eicosatrienoic acid ethanolamide
5S,14R-LXB4	5S,14R-lipoxin B4
5S,6R-LXA4	5S,6R-lipoxin A4
5S,6S-LXA4	5S,6S-lipoxin A4
6-keto-PGF1a	6-keto-prostaglandin F1alpha
6-trans-LTB4	6-trans-leukotriene B4
6,15-diketo-13,14-dihydro-PGF1a	6,15-diketo-13,14-dihydro-prostaglandin F1alpha
7-HDoHE	7-hydroxy-docosahexaenoic acid
7,17-hydroxy-DPA	7,17-dihydroxy-docosapentaenoate
8-HDoHE	8-hydroxy-docosahexaenoic acid
8-HETE	8-hydroxy-eicosatetraenoic acid
8-iso-13,14-dihydro-15-keto-PGF2a	8-iso-13,14-dihydro-15-keto-prostaglandin F2alpha
8-iso-15-keto-PGF2a	8-iso-15-keto-prostaglandin F2alpha
8-iso-15(R)-PGF2a	8-iso-15(R)-prostaglandin F2alpha
8-iso-PGA1	8-iso-prostaglandin A1
8-iso-PGA2	8-iso-prostaglandin A2
8-iso-PGE1	8-iso-prostaglandin E1
8-iso-PGE2	8-iso-prostaglandin E2
8-iso-PGF1a	8-iso-prostaglandin F1alpha
8-iso-PGF2a	8-iso-prostaglandin F2alpha
8-iso-PGF3a	8-iso-prostaglandin F3alpha
8,12-iso-iPF2a-VI-1,5-lactone	8,12-iso-isoprostane F2 alpha-VI-1,5-lactone
8,15-DiHETE	8,15-dihydroxy-eicosatetraenoic acid
8,9-DHET	8,9-dihydroxy-eicosatrienoic acid
8,9-EET	8,9-epoxy-eicosatrienoic acid
8,9-EET-EA	8,9-epoxy-eicosatrienoic acid ethanolamide
9-HETE	9-hydroxy-eicosatetraenoic acid
9-HODE	9-hydroxy-octadecadienoic acid
9-HOTrE	9-hydroxy-octadecatrienoic acid
9-HpODE	9-hydroperoxy-octadecadienoic acid
9-KODE	9-oxo-octadecadienoic acid
9,10-DiHOME	9,10-dihydroxy-octadecenoic acid
9,10-EpOME	9,10-epoxy-octadecenoic acid
10-HDoHE	10-hydroxy-docosahexaenoic acid
10,17-DiHDoHE	10,17-dihydroxy-docosahexaenoic acid
11-beta-13,14-dihydro-15-keto-PGF2a	11-beta-13,14-dihydro-15-keto-prostaglandin F2alpha
11-beta-PGF2a	11-beta-prostaglandin F2alpha
11-dehydro-TXB2	11-dehydro-thromboxane B2
11-HDoHE	11-hydroxy-docosahexaenoic acid
11-HETE	11-hydroxy-eicosatetraenoic acid
11-trans-LTC4	11-trans-leukotriene C4
11-trans-LTE4	11-trans-leukotriene E4
11,12-DHET	11,12-dihydroxy-eicosatrienoic acid
11,12-EET	11,12-epoxy-eicosatrienoic acid
11,12-EET-EA	11,12-epoxy-eicosatrienoic acid ethanolamide
12-HEPE	12-hydroxy-eicosapentaenoic acid
12-HETE	12-hydroxy-eicosatetraenoic acid
12-HHT	12-hydroxy-heptadecatrienoic acid
12-HpEPE	12-hydroperoxy-eicosapentaenoic acid
12-HpETE	12-hydroperoxy-eicosatetraenoic acid
12-KETE	12-oxo-eicosatetraenoic acid
12-keto-LTB4	12-keto-leukotriene B4
12,13-DiHOME	12,13-dihydroxy-octadecenoic acid
12,13-EpOME	12,13-epoxy-octadecenoic acid
13-HDoHE	13-hydroxy-docosahexaenoic acid
13-HODE	13-hydroxy-octadecadienoic acid
13-HOTrE	13-hydroxy-octadecatrienoic acid
13-HpODE	13-hydroperoxy-octadecadienoic acid
13-KODE	13-keto-octadecadienoic acid
13,14-dihydro-15-keto PGJ2	13,14-dihydro-15-keto prostaglandin J2
13,14-dihydro-15-keto-PGD2	13,14-dihydro-15-keto-prostaglandin D2
13,14-dihydro-15-keto-PGE2	13,14-dihydro-15-keto-prostaglandin E2
13,14-dihydro-15-keto-PGF2a	13,14-dihydro-15-keto-prostaglandin F2alpha
13,14-dihydro-15-keto-tetranor-PGD2	13,14-dihydro-15-keto-tetranor-prostaglandin D2
13,14-dihydro-15-keto-tetranor-PGE2	13,14-dihydro-15-keto-tetranor-prostaglandin E2
13,14-dihydro-15-keto-tetranor-PGF1a	13,14-dihydro-15-keto-tetranor-prostaglandin F1alpha
13,14-dihydro-15-keto-tetranor-PGF1b	13,14-dihydro-15-keto-tetranor prostaglandin F1beta
14-HDoHE	14-hydroxy-docosahexaenoic acid
14,15-DHET	14,15-dihydroxy-eicosatrienoic acid
14,15-DiHETE	14,15-dihydroxy-eicosatetraenoic acid
14,15-EET	14,15-epoxy-eicosatrienoic acid
14,15-EET-EA	14,15-epoxy-eicosatrienoic acid ethanolamide
14,15-LTC4	14,15-leukotriene C4
14,15-LTE4	14,15-leukotriene E4
15-deoxy-delta-12,14-PGJ2	15-deoxy-delta-12,14-prostaglandin J2
15-HEDE	15-hydroxy-eicosadienoic acid
15-HEPE	15-hydroxy-eicosapentaenoic acid
15-HETE	15-hydroxy-eicosatetraenoic acid
15-HETrE	15-hydroxy-eicosatrienoic acid
15-HpEPE	15-hydroperoxy-eicosapentaenoic acid
15-HpETE	15-hydroperoxy-eicosatetraenoic acid
15-KEDE	15-oxo-eicosadienoic acid
15-keto-PGE2	15-keto-prostaglandin E2
15-keto-PGF2a	15-keto-prostaglandin F2alpha
16-HDoHE	16-hydroxy-docosahexaenoic acid
16-HETE	16-hydroxy-eicosatetraenoic acid
17-HDoHE	17-hydroxy-docosahexaenoic acid
17-HETE	17-hydroxy-eicosatetraenoic acid
17,18-DiHETE	17,18-dihydroxy-eicosatetraenoic acid
17,18-EpETE	17,18-epoxy-eicosatetraenoic acid
18-carboxy-dinor-LTB4	18-carboxy-dinor-leukotriene B4
18-HEPE	18-hydroxy-eicosapentaenoic acid
18-HETE	18-hydroxyicosa-tetraenoic acid
19-HETE	19-hydroxy-eicosatetraenoic acid
1a1b-dihomo-PGF2a	1a,1b-dihomo-prostaglandin F2alpha
2,3-dinor-8-iso-PGF2a	2,3-dinor-8-iso-prostaglandin F2alpha
20-carboxy-AA	20-carboxy-arachidonic acid
20-carboxy-LTB4	20-carboxy-leukotriene B4
20-HDoHE	20-hydroxy-docosahexaenoic acid
20-HETE	20-hydroxy-eicosatetraenoic acid
20-hydroxy-LTB4	20-hydroxy-leukotriene B4
20-hydroxy-PGE2	20-hydroxy-prostaglandin E2
20-hydroxy-PGF2a	20-hydroxy-prostaglandin F2alpha
AA	arachidonic acid
AEA	arachidonylethanolamide
Azelaoyl-PAF	azelaoyl-platelet-activating factor
DHA	docosahexaenoic acid
EPA	eicosapentaenoic acid
iPF2a-IV	isoprostane F2 alpha-IV
LTB4	leukotriene B4
LTB4-EA	leukotriene B4 ethanolamide
LTC4	leukotriene C4
LTD4	leukotriene D4
LTE4	leukotriene E4
LTF4	leukotriene F4
LXA5	lipoxin A5
Lyso-PAF	lyso-platelet-activating factor
Maresin1	maresin1
N-acetyl-LTE4	N-acetyl leukotriene E4
OEA	oleoylethanolamine
PAF	platelet-activating factor
PGA1	prostaglandin A1
PGA2	prostaglandin A2
PGB2	prostaglandin B2
PGD1	prostaglandin D1
PGD2	prostaglandin D2
PGD2-EA	prostaglandin D2 ethanolamide
PGD3	prostaglandin D3
PGE1	prostaglandin E1
PGE1-EA	prostaglandin E1 ethanolamide
PGE2	prostaglandin E2
PGE2-EA	prostaglandin E2 ethanolamide
PGE3	prostaglandin E3
PGF2a	prostaglandin F2alpha
PGF2a-EA	prostaglandin F2alpha ethanolamide
PGF3a	prostaglandin F3alpha
PGJ2	prostaglandin J2
PGK2	prostaglandin K2
Resolvin D1	resolvin D1
Resolvin D2	resolvin D2
tetranor-PGDM	tetranor-prostaglandin D metabolite
tetranor-PGEM	tetranor-prostaglandin E metabolite
tetranor-PGFM	tetranor-prostaglandin F metabolite
TXB1	thromboxane B1
TXB2	thromboxane B2
TXB3	thromboxane B3
